# SHED-Dependent Oncogenic Signaling of the PEAK3 Pseudo-Kinase

**DOI:** 10.3390/cancers13246344

**Published:** 2021-12-17

**Authors:** Youcef Ounoughene, Elise Fourgous, Yvan Boublik, Estelle Saland, Nathan Guiraud, Christian Recher, Serge Urbach, Philippe Fort, Jean-Emmanuel Sarry, Didier Fesquet, Serge Roche

**Affiliations:** 1 CRBM, University Montpellier, CNRS, Equipe Labellisée Ligue Contre le Cancer, F-34000 Montpellier, France; youcef.ounoughene@crbm.cnrs.fr (Y.O.); elise.fourgous@crbm.cnrs.fr (E.F.); yvan.boublik@crbm.cnrs.fr (Y.B.); philippe.fort@crbm.cnrs.fr (P.F.); 2 CRCT, INSERM, CNRS, University of Toulouse, Equipe Labellisée Ligue Contre le Cancer, F-31037 Toulouse, France; estelle.saland@inserm.fr (E.S.); nathan.guiraud@inserm.fr (N.G.); christian.recher@inserm.fr (C.R.); jean-emmanuel.sarry@inserm.fr (J.-E.S.); 3 IGF, CNRS, INSERM, University Montpellier, F-34000 Montpellier, France; serge.urbach@igf.cnrs.fr

**Keywords:** pseudo-kinase, oncogene, cell signaling, cell growth and migration, tyrosine kinase, AKT signaling

## Abstract

**Simple Summary:**

The human kinome is composed of about 50 pseudo-kinases with unclear function, because they are predicted to be catalytically inactive; however, they are shown to play an important role in cancer, similar to active kinases. Understanding how these pseudo-kinases promote tumor formation despite their catalytic inactivity is a great challenge, which may lead to innovative anti-cancer therapies. The PEAK1 and 2 pseudo-kinases have emerged as important components of the protein tyrosine kinase pathway implicated in cancer progression. They can signal using a scaffolding mechanism via a conserved split helical dimerization (SHED) module. In this study, we uncovered a similar SHED-dependent oncogenic activity for PEAK3, a recently discovered new member of this family. We also show that this new signaling mechanism may be implicated in acute myeloid leukemia.

**Abstract:**

The PEAK1 and Pragmin/PEAK2 pseudo-kinases have emerged as important components of the protein tyrosine kinase pathway implicated in cancer progression. They can signal using a scaffolding mechanism that involves a conserved split helical dimerization (SHED) module. We recently identified PEAK3 as a novel member of this family based on structural homology; however, its signaling mechanism remains unclear. In this study, we found that, although it can self-associate, PEAK3 shows higher evolutionary divergence than PEAK1/2. Moreover, the PEAK3 protein is strongly expressed in human hematopoietic cells and is upregulated in acute myeloid leukemia. Functionally, PEAK3 overexpression in U2OS sarcoma cells enhanced their growth and migratory properties, while its silencing in THP1 leukemic cells reduced these effects. Importantly, an intact SHED module was required for these PEAK3 oncogenic activities. Mechanistically, through a phosphokinase survey, we identified PEAK3 as a novel inducer of AKT signaling, independent of growth-factor stimulation. Then, proteomic analyses revealed that PEAK3 interacts with the signaling proteins GRB2 and ASAP1/2 and the protein kinase PYK2, and that these interactions require the SHED domain. Moreover, PEAK3 activated PYK2, which promoted PEAK3 tyrosine phosphorylation, its association with GRB2 and ASAP1, and AKT signaling. Thus, the PEAK1-3 pseudo-kinases may use a conserved SHED-dependent mechanism to activate specific signaling proteins to promote oncogenesis.

## 1. Introduction

The human kinome includes >50 pseudo-kinases that are predicted to be catalytically inactive due to the lack of important residues required for full enzymatic activity [1]. Their mechanistic role in cell signaling remains unclear, but recent structural analyses suggest a scaffolding or allosteric activity by docking additional kinases for efficient protein phosphorylation [1,2,3]. Moreover, some pseudo-kinases have retained active kinase activity through an unconventional mechanism of protein phosphorylation [1,2,3]. These atypical kinases have gained recent interest because they play important roles as active protein kinases in human cancer. For instance, many pseudo-kinases, such as HER3, TRIBL, and JAK2 (named JH2), are overexpressed or mutated in various cancer types and contribute to tumor progression [1,2,3].

The PEAK pseudo-kinases comprise PEAK1, Pragmin/PEAK2, and chromosome 19 open reading frame 35 (C19orf35)/PEAK3 [4,5]. They include a variable N-terminal sequence with specific interaction motifs (e.g., SH2 and SH3 binding sites) followed by a shared pseudo-kinase domain. Pragmin/PEAK2 (hereafter PEAK2), the family founder, was originally identified as an effector of the small GTPase Rnd2 to mediate cell contraction [6]. PEAK1 was identified later as a novel cytoskeletal-associated atypical kinase [7]. More recently, using an in-silico sequence–structure analysis, we identified C19orf35, as an additional family member, hence the name PEAK3 [8]. PEAK1/2 are essential components of tyrosine kinase (TK) signaling, leading to cell growth and migration [4,5]. Mechanistically, through tyrosine phosphorylation they allow the recruitment of important intracellular signaling effectors, such as GRB2 and SHC [7,9,10,11]. We and other groups have described PEAK1/2 pro-tumor activity [4,5]. Specifically, these pseudo-kinases are overexpressed in many epithelial cancer types and contribute to aggressive tumor progression by promoting cancer cell growth and invasion [7,10,11,12,13,14,15,16,17]. Importantly, their aberrant expression in tumors may deregulate cancer cell adhesive properties and enable metastatic progression. 

Crystallographic studies revealed an important mechanism by which PEAK1/2 pseudo-kinases may regulate oncogenic pathways [8,18,19]. In addition to a classical protein kinase fold harboring an occluded ATP binding site, three crystal structures revealed an original dimerization domain, i.e., split helical dimerization (SHED) [8,18,19]. This conserved module comprises a long helix N-terminal to the pseudo-kinase domain and three additional helices from the C-terminal extension that form an “XL”-shaped helical bundle. The SHED-based dimerization mechanism regulates PEAK1/2 homo- and hetero-dimerization that might influence PEAK-signaling specificity [20]. However, it is not known whether the SHED domain defines an essential mechanism of PEAK signaling.

**Figure 1:**
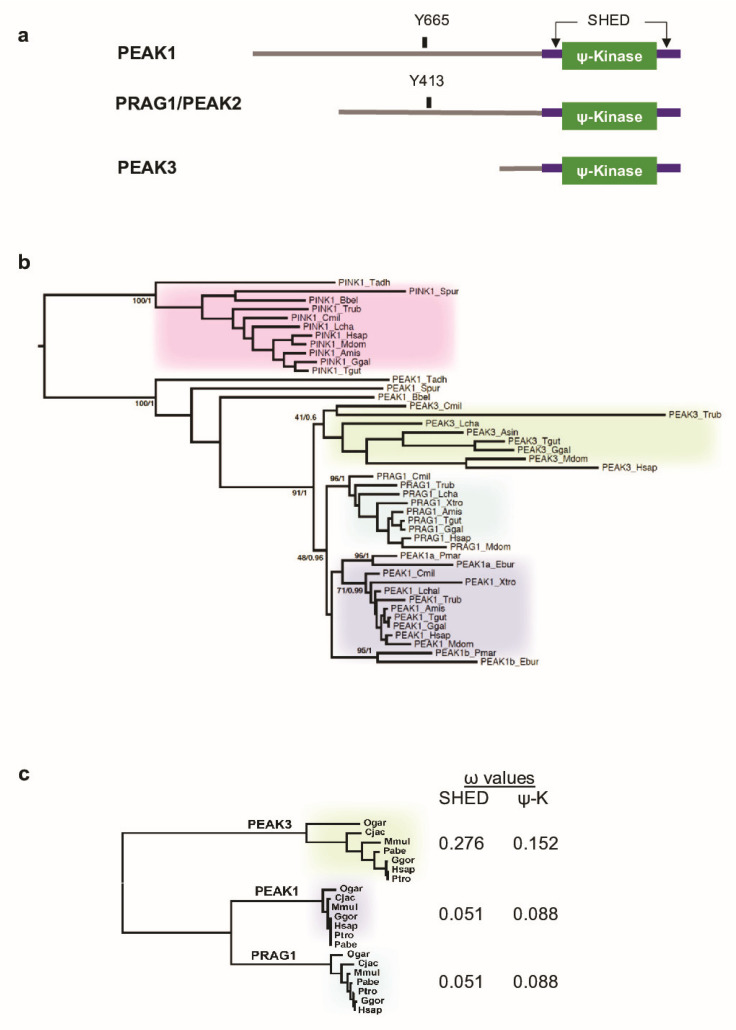
Phylogenetic analysis of PEAK pseudo-kinases. (**a**) Modular structure of PEAK1-3 pseudo-kinases. The split helical dimerization (SHED) module (made of αN, αJ, αK, and αN helices) (purple), the pseudo-kinase (φ-Kinase) (green), and the main known tyrosine phosphorylation sites are highlighted. (**b**) Phylogenetic cladogram of PEAK proteins. The tree was deduced from a multiple amino acid sequence alignment of kinase and pseudo-kinase domains and processed by PhyML and MrBayes analysis. PTEN-induced kinases (PINK) were included as an external group to root the tree. Only supports for key phylogenetic nodes are indicated (bootstrap proportion/posterior probability). Species and sequences (accession numbers) used for the alignment are listed in Table S1. (**c**) Analysis of the non-synonymous to silent substitution ratios (ω) of the SHED and pseudo-kinase (ΨK) domains in primate PEAKs. The nucleic sequences of the domains were aligned based on translation and processed with codeml to estimate the ω values. The phylogenetic tree on the left was generated by PhyML analysis of a multiple alignment of the ΨK amino acid sequences. Species and sequences (accession numbers) used for the alignment are listed in Table S1.

PEAK3 was identified as a member of the PEAK family, due to the fact that its peptide sequence predicts a similar degraded ATP-binding site in the TK flanked by the SHED module [8] (Figure 1a). Moreover, PEAK3 self-associates in a SHED-dependent manner [21], but its biological activity is poorly characterized. Recently, Lopez et al. reported that PEAK3 expression can prevent CRKII-dependent membrane ruffling [21], suggesting a negative function in cell migration, dissimilar to PEAK1/2. In this study, we investigated PEAK3’s functional role in cancer. We found that PEAK3 promotes cancer cell growth and migration. Moreover, the SHED module has an important function in these effects through a unique PEAK3/PYK2/AKT signaling cascade. Our findings support the existence of a common SHED-dependent mechanism of PEAK1-3’s oncogenic activity, mediated by the recruitment of a unique set of signaling proteins.

## 2. Results

### 2.1. PEAK3 Evolutionary Conservation

Unlike the human genome (three PEAK genes) (Figure 1a), we found a single PEAK gene in the genomes of early metazoan species, from placozoans (*Trichoplax adherens*) to early chordates (the lancelet *Branchiostoma*) [21] (Figure 1b) [8]. To better understand the PEAK family ontology, we searched for homologous sequences in key evolutionary species. We identified two PEAK members in cyclostomes (the lamprey, *Petromyzon marinus*, and the hagfish, *Eptatretus burgeri*) and three members in cartilaginous fishes (e.g., the elephant shark, *Callorhinchus milii*), bony fishes (e.g., *Takifugu rubripes*), and the coelacanth, *Latimeria chalumnae*. We then extracted the three homologous sequences in additional Tetrapoda species and performed a clustering analysis of MSAs using two probabilistic algorithms (maximum-likelihood and Bayesian). We used the related PTEN-induced kinases (PINK) as the external group to root the tree. The clustering tree globally followed the species phylogeny and showed the presence of a well-supported node leading to three PEAK1-3 clusters in vertebrates (Figure 1b). The branching of the three nodes suggests that a first duplication led to PEAK1/2 and PEAK3, followed by the PEAK1/2 duplication into PEAK1 and PEAK2. This shows that PEAK1-3 emerged at the onset of vertebrates and not in Sauropsida as previously proposed [21]. The presence of only PEAK1-like proteins in cyclostomes suggests that they lost PEAK2 and PEAK3. However, the lower conservation of PEAK3, as indicated by the longer internal branches, may have blurred the branching of the three groups to such an extent that the exact duplication order can no longer be deduced.

To firmly establish the lower conservation of PEAK3 compared with PEAK1/2, we examined the selective constraints exerted on the SHED and pseudo-kinase (ΨK) domains. We calculated the dN/dS ω ratio from a MSA of seven primate species that covered 74 MY of evolution. Neutral selection is associated with ω values close to 1, while values below or above 1 indicate purifying and diversifying selection, respectively. The selection exerted on the SHED and ΨK domains was lower for PEAK3 than for PEAK1 and PEAK2 (Figure 1c), which is in agreement with the difference in branch length. The lowest PEAK3 conservation is also consistent with with the observation that the corresponding gene was lost in several taxa, for instance Myomorpha in rodents (Table S1). The maintenance of duplicated genes is often associated with neo- or sub-functionalization (i.e., the acquisition of new functions or differential tissue expression, respectively) [22]. Then, we investigated *PEAK1–3* mRNA expression using the Human Protein Atlas [23] and found that *PEAK1* was ubiquitously expressed, particularly in spleen; *PEAK2* expression was more restricted, with the highest expression in cerebellum; and *PEAK3* was nearly exclusively expressed in lymphoid tissues (spleen, lymph nodes) and granulocytes (Figure S1). Overall, these data indicate that, although PEAK3 is much less evolutionary conserved than its paralogues PEAK1 and PEAK2, it underwent purifying selection indicative of a physiological function, possibly in blood cells.

### 2.2. PEAK3 Overexpression in Acute Myeloid Leukemia

**Figure 2:**
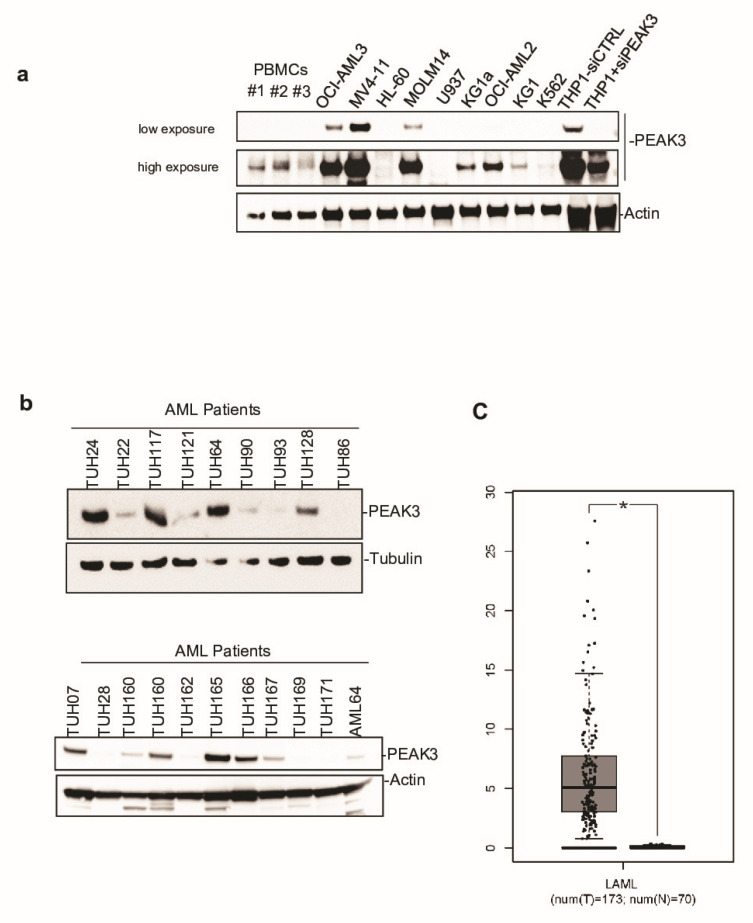
PEAK3 expression in AML. (**a**) PEAK3 protein expression in the indicated blood cell lines revealed by western blotting with an anti-PEAK3 antibody. Antibody specificity was confirmed using THP1 cells transfected with a siRNA targeting PEAK3. Both low and longer exposure are shown. PBMCs: peripheral blood mononuclear cells (*n* = 2). (**b**) PEAK3 expression in AML patient samples. The level of actin or tubulin in also shown (*n* = 2). (**c**) PEAK3 upregulation in AML patient samples. The panel shows the relative level of PEAK3 transcripts in 173 AML samples (T) and 70 normal samples (N), (gepia.cancer-pku.cn). * *p* < 0.05 (Student’s *t* test).

Next, to investigate PEAK3 protein expression in human cells, we generated a polyclonal antibody against full-length recombinant PEAK3. This antibody could detect PEAK3 in lysates from PEAK3-overexpressing U2OS and HeLa cells in western blotting (Figure S2a,b). It also recognized an endogenous protein of about 50 KDa in lysates of THP1 (AML) cells (Figure S2c). We confirmed that this protein was PEAK3, because the 50 KDa band disappeared upon siRNA-mediated PEAK3 knockdown (Figure S2c). We then used this antibody to assess PEAK3 expression in various human cell lines. We did not detect endogenous PEAK3 in the mesenchymal and epithelia cell lines tested (Figure S2c), but only in hematopoietic cell lines, specifically in AML cell lines (Figure 2a and Figure S2c). Consistent with this finding, we observed elevated PEAK3 protein expression in 40% of the tested AML patient samples (8/20) (Figure 2b and Table S2). Analysis of the *PEAK3* transcript level from TCGA transcriptomic data (gepia.cancer-pku.cn accessed on 2 January 2021) confirmed that *PEAK3* was upregulated in AML (Figure 1c), particularly in the M4 and M5 subtypes (Figure S3a). PEAK3 aberrant expression was preferentially associated with specific oncogenic mutations, i.e., DNTM3A mutations, but not with FLT3-TD mutations (Figure 2b and Figure S3b). Our findings indicate that PEAK3 is expressed in hematopoietic cells and that its expression can be deregulated in AML, suggesting a pro-tumor function.

### 2.3. SHED-Dependent Oncogenic Activity of PEAK3

**Figure 3:**
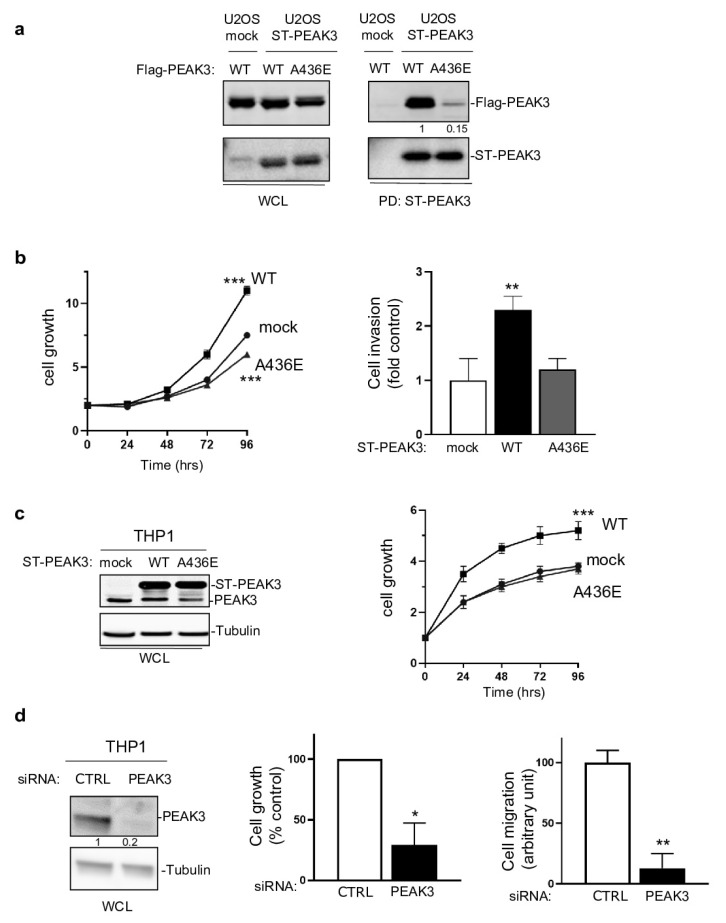
SHED-dependent PEAK3 oncogenic activity. (**a**) SHED-dependent PEAK3 self-association. Co-precipitation of Srep-Tag-PEAK3 (ST-PEAK3) on Strep-Tactin magnetic beads (PD: pull down) with FLAG-PEAK3 and A436E mutant in U2OS cells stably expressing an empty vector (mock) or ST-PEAK3 that were transfected with indicated FLAG-PEAK3 construct. The level of PEAK3 in whole-cell lysates (WCL) is also shown (*n* = 2). (**b**) PEAK3 expression promotes U2OS cell growth in a SHED-dependent manner. Cell growth over time (fold vs. control) (left) and cell invasion in Boyden chambers coated with Matrigel (right) in U2OS cells that express the indicated PEAK3 constructs described in panel A (WT: wild type PEAK3; A436E: PEAK3 with an inactive SHED domain). (**c**) ST-PEAK3 level (left) and cell growth over time (fold vs. control) (left) in THP1 cells that express the indicated ST-PEAK3 constructs. (**d**) PEAK3 knockdown (left) reduces THP1 cell growth vs. control cells (cell growth monitored for 3 days) and migration in Boyden chambers coated with an endothelial cell monolayer. Mean ± SD are shown (*n* = 3); * *p* < 0.05; ** *p* < 0.01; *** *p* < 0.001 (Student’s *t* test).

We then investigated whether PEAK3 had pro-tumor functions. PEAK3 overexpression in U2OS cells (Figure 3a) and in THP1 cells (Figure 3c) increased cell growth and invasion in Matrigel-coated Boyden chambers (Figure 3b). These effects were abrogated in both cell lines by overexpression of PEAK3 harboring the A436E mutation in the C-terminal extension of the SHED module (Figure 3a–c), which destabilized its dimerization (Figure 3a and Figure S4a) [21]. Similarly, siRNA-mediated *PEAK3* silencing in THP1 cells reduced their growth and migration in Boyden chambers coated with an endothelial cell monolayer (Figure 3d and Figure S5). These findings indicate that, in human cancer cells, PEAK3 displays oncogenic activity that requires an intact SHED module. As PEAK1/2 localize at focal adhesions (FAs) [7,13], we investigated PEAK3 localization by direct fluorescence analysis of U2OS cells that stably express GFP-PEAK3 and demonstrated PEAK3 localization at FAs (Figure S6a) and partial co-localization with paxillin (Figure S6a). We confirmed PEAK3 localization at FAs by indirect immunofluorescence using an anti-HA antibody in U2OS cells that stably express HA-ST-PEAK3 (Figure S6b). Additionally, both imaging methods showed a strong PEAK3 nuclear localization (Figure S6). Conversely, the PEAK3 A436E mutant did not display FA localization, but was still localized in the nucleus (Figure S6a,b). These observations indicate that PEAK3 localizes at FAs such as PEAK1/2, and that this requires a SHED dimeric module.

### 2.4. PEAK3 Activates AKT Signaling

**Figure 4:**
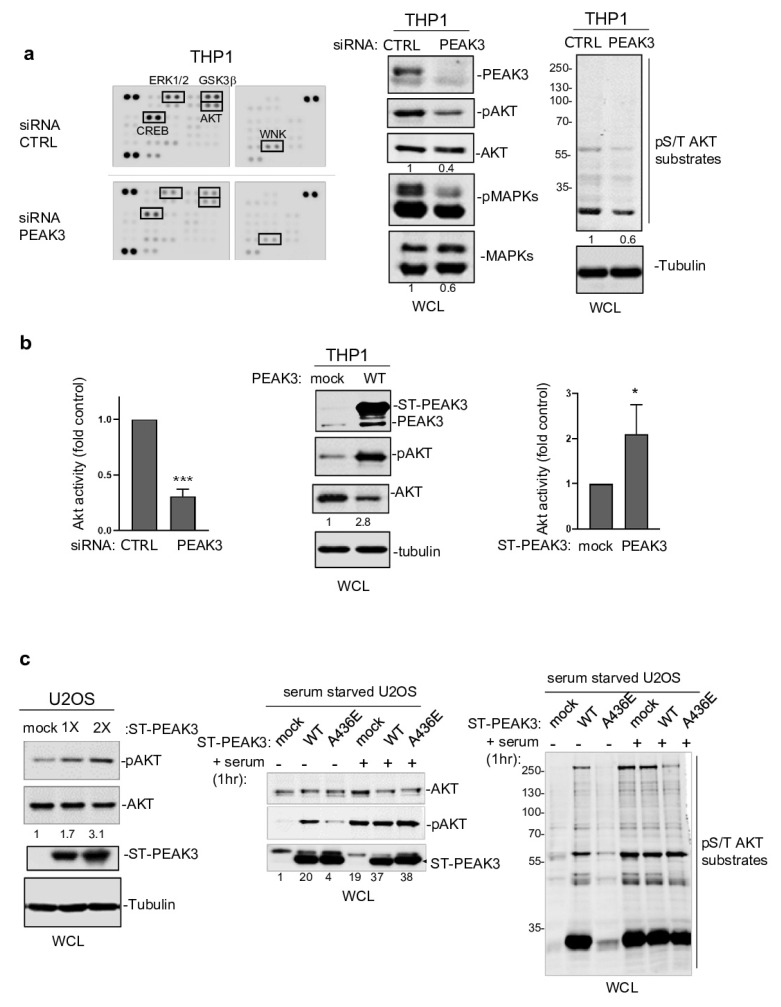
PEAK3 activates AKT signaling in the absence of growth factors. (**a**) (left) Phospho-kinase array analysis of THP1 cells transfected with siRNA control (CTRL) or siRNA against PEAK3. The phospho-signal reduction in the siRNA PEAK3 condition is highlighted (i.e., pT202/Y204 ERK1/2; pS21/9 GSK3β; pS133 CREB; pS473 AKT; and pT60 WNK). (right) Western blot analysis of MAPK, AKT activity, and signaling (pS/T AKT substrates level). Tubulin level and relative AKT and MAPK activity is shown. (**b**) (left) Quantification of AKT inhibition upon PEAK3 silencing (mean ± SD; *n* = 3); *** *p* < 0.001 (Student’s *t* test) (bottom). PEAK3 overexpression increases AKT activity in THP1 cells. A representative western blot (middle) and relative quantification of AKT activation (right) (mean ± SD; *n* = 3); * *p* < 0.05 (Student’s *t* test). (**c**) PEAK3 expression induces AKT signaling in U2OS cells, even in the absence of growth factors. (left) AKT activation and its relative quantification upon transient PEAK3 expression (1×: 1 μg, 2×: 2 μg of PEAK3 construct transfected) (*n* = 2). SHED-dependent PEAK3 activation of AKT signaling in the absence of growth factors. U2OS cells that stably express WT or A436E PEAK3 were serum-starved for >20 h and stimulated or not with 10% FCS for 1 h. Then, the levels of AKT, phosphorylated AKT (middle), and pS/T AKT substrates were assessed (right) (*n* = 2).

Next, to determine PEAK3 oncogenic signaling, we probed a phospho-kinase antibody array from THP1 cells transfected with a control siRNA or a siRNA targeting *PEAK3* (Figure 4a). We found that PEAK3 downregulation affected the activating phosphorylation of adhesive TKs of the Src (SFK) and FAK families (Figure S7a) and also reduced MAPK and AKT activating phosphorylations (Figure 4a and Figure S7a). We confirmed these results using western blotting (Figure 4a, right panel). *PEAK3* silencing also reduced phosphorylation of the probed AKT substrates (i.e., GSK-3 alpha/beta, PRAS40, and CREB), suggesting that this pseudo-kinase is a novel upstream regulator of this pathway (Figure S7a). Consistently, AKT activity (i.e., pS473-AKT level) increased approximately 2-fold upon PEAK3 overexpression and reduced 3-fold upon PEAK3 knockdown (Figure 4b). We obtained similar results in PEAK3-overexpressing U2OS cells, suggesting that PEAK3 might be a general inducer of AKT signaling (Figure 4c). PEAK3 overexpression induced a modest increase in AKT signaling (i.e., AKT activity and AKT substrate phosphorylation). Conversely, PEAK3 strongly activated AKT in serum-starved conditions (Figure 4c). We did not observe this effect with the PEAK3 A436E mutant, further supporting the essential role of the SHED module in PEAK3 signaling (Figure 4c). PEAK3–AKT signaling was abrogated by pharmacological inhibition of PI3K activity (Figure S7b), suggesting that PEAK3 is upstream to PI3K. Collectively, these data indicate that PEAK3 induces PI3K/AKT signaling, even in the absence of growth factors.

### 2.5. SHED-Dependent PEAK3 Scaffolding Activity

**Figure 5:**
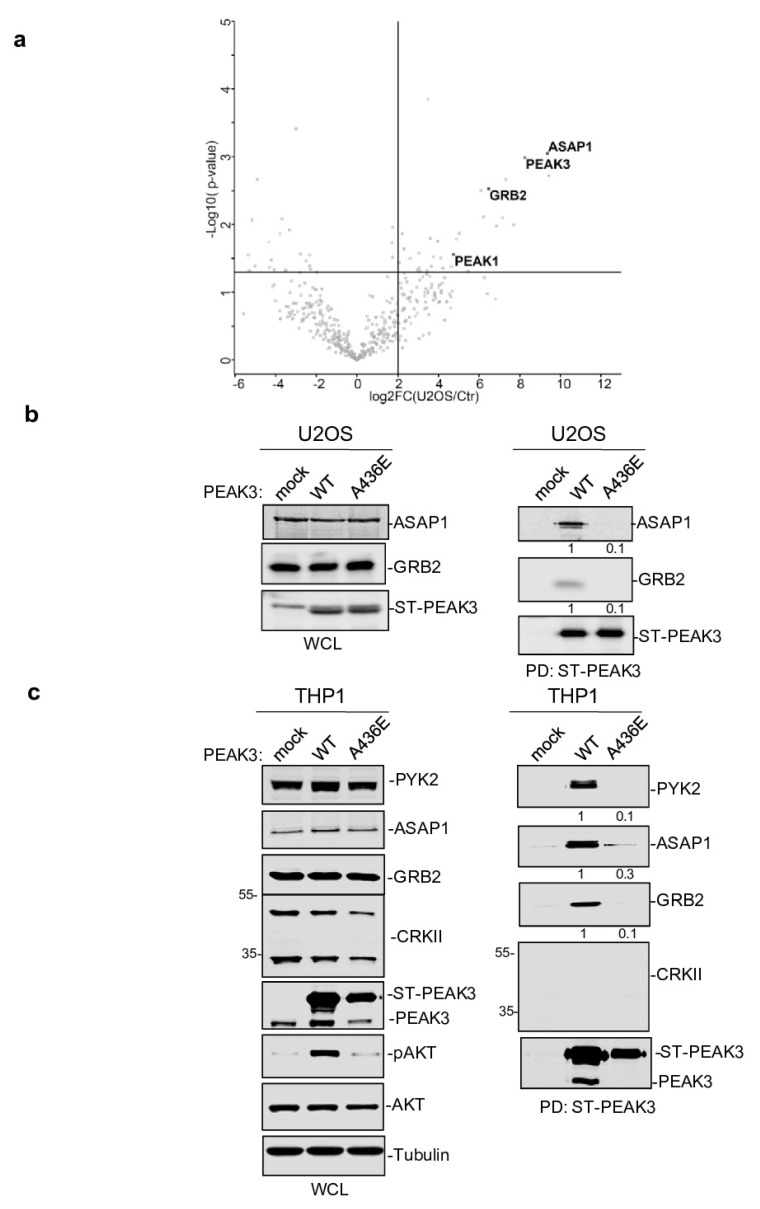
PEAK3 interactomics identified GRB2, ASAP1/2, and PYK2 as SHED-dependent binders. (**a**) Interactome of PEAK3 in U2OS cells represented as volcano plots of the MS data (*n* = 3). (**b**) and (**c**) Validation by western blot analysis of PEAK3 interaction with the indicated signaling proteins in U2OS (**b**) and THP1 cells (**c**) (*n* = 2). The relative levels of PYK2, ASAP1, and GRB2 associated with ST-PEAK3 WT and AE mutant are shown.

To further elucidate the mechanism by which PEAK3 induces AKT signaling, we performed PEAK3 interactomic analyses of U2OS, HeLa, and THP1 cells that stably express ST-PEAK3 (Figure 5a and Figure S4b and Table S3). Label-free LC-MS/MS analysis of ST-PEAK3 complexes purified on streptavidin beads identified many signaling proteins in the three PEAK3 interactomes, including GRB2, ADP-ribosylation factor (ARF) GTPase-activating proteins ASAP1 and 2, and another member of the PEAK family (i.e., PEAK1 in the HeLa and U2OS cell interactomes and PEAK2 in the THP1 cell interactome). This proteomic analysis also identified many 14-3-3 proteins, which regulate phospho-dependent signaling activity [24] and voltage-dependent anion-selective channel proteins (i.e., VDAC1-3), which regulate cell volume and apoptosis (Table S3) [25]. Additionally, we detected the FAK-like TK PYK2 in the PEAK3 interactome of THP1 cells (Table S3). We next confirmed some of these findings using western blotting (Figure 5b,c). Although the CRKII cytoskeletal proteins were previously reported as PEAK3 binders [21], we could not detect any endogenous CRKII protein associated with PEAK3 in our proteomic and biochemical analyses (Figure 5c and Table S3). All these interactions required an intact SHED module (Figure 5c), suggesting that PEAK3 dimerization is essential for interaction with these signaling proteins. As ASAP1/2, VDACs, and PYK2 have not been detected in the previously reported PEAK1 and PEAK2 interactomes [8,20], PEAK1-3 may recruit a unique set of signaling proteins to induce intracellular signaling.

### 2.6. PEAK3 Activates PYK2 to Promote AKT Signaling

**Figure 6:**
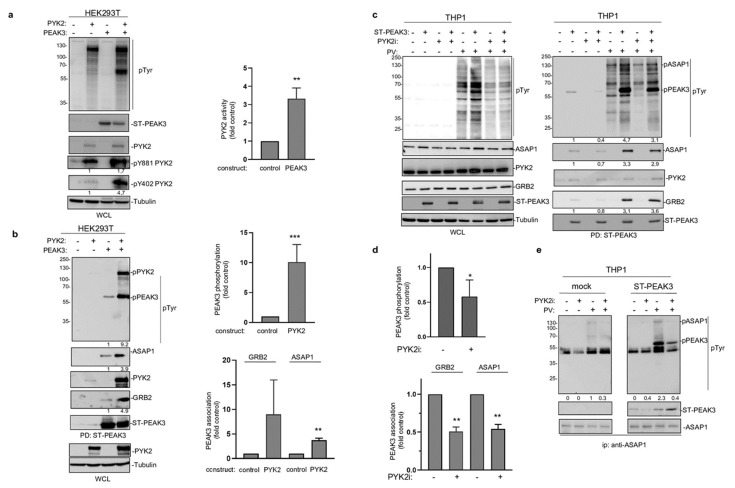
PEAK3 activates PYK2 signaling. (**a**) PYK2 activation by PEAK3 in HEK293T cells. The levels of protein tyrosine phosphorylation, PEAK3, PYK2, and phosphorylated PYK2 (pPYK2, pTyr402, and pTyr881) were assessed in cells transfected with the indicated constructs for 40 h (left). Relative quantification of PYK2 activation by PEAK3 (mean ± SD; *n* = 3); ** *p* < 0.01 (Student’s *t* test) (right). (**b**) PEAK3 tyrosine phosphorylation and its association with GRB2 and ASAP1 by PYK2 in HEK293T cells. The level-indicated proteins and their tyrosine phosphorylation in the PEAK3 pull-down were assessed in cells transfected with indicated constructs for 40 h. Relative quantification of PEAK3 tyrosine phosphorylation and its association with GRB2 and ASAP1 (mean ± SD; *n* = 3); ** *p* < 0.01, *** *p* < 0.001 (Student’s *t* test) (right). (**c**) PEAK3 tyrosine phosphorylation by PYK2 in THP1 cells. The pull-down of PEAK3 and its associated proteins were assessed in THP1 cells overexpressing PEAK3 and treated or not with pervanadate (PV) (0.1 μM for 15 min) or PYK2i (1 μM for 2h) as indicated. (**d**) Relative quantification of PEAK3 tyrosine phosphorylation and its association with GRB2 and ASAP1 in THP1 cells (mean ± SD; *n* = 3); * *p* < 0.05, ** *p* < 0.01 (Student’s *t* test) (right). (**e**) Increased ASAP1 tyrosine phosphorylation by PEAK3 expression in a PYK2-dependent manner. Immunoprecipitated ASAP1 tyrosine phosphorylation and its association with ST-PEAK3 from indicated THP1 cells treated as described in Figure 6c (*n* = 2).

The fact that PEAK3 interacts with PYK2 suggests that, like PEAK2 [4,8], PEAK3 may activate specific TKs to mediate phospho-tyrosine signaling. We tested this hypothesis by co-expressing PEAK3 and PYK2 in HEK293T cells. While PEAK3 alone did not clearly affect protein tyrosine phosphorylation, PEAK3 robustly activated ectopic PYK2, as measured on the phosphorylation level of regulatory tyrosine 402 and 881 (Figure 6a,c) [26]. Interestingly, PYK2 activation was accompanied by an increase in cellular protein tyrosine phosphorylation, including a 50 KD and 120 KDa band (Figure 6a). Pull-down experiments next identified PEAK3 and PYK2 some of these tyrosine phosphorylated proteins (Figure 6b). Similar results were obtained in THP1cells where tyrosine phosphorylation of overexpressed PEAK3 was reduced upon acute pharmacological inhibition of PYK2 (PYK2i) (Figure 6c,d). Additionally, the pharmacological inhibition of protein tyrosine phosphatases (pervanadate) largely increased PEAK3 tyrosine phosphorylation (Figure 6c), suggesting the existence of regulatory protein tyrosine phosphatases. Consequently, PEAK3 tyrosine phosphorylation by PYK2 increased its association with GRB2 and ASAP1 (Figure 6b), while PYK2i reduced this effect (Figure 6c,d). ASAP1 was previously identified as a PYK2 substrate [27] and, consistent with our findings, overexpressed PEAK3 increased ASAP1 tyrosine phosphorylation in a PYK2-dependent manner (Figure 6e). Finally, this molecular process required an intact SHED module (Figure S8), suggesting that PEAK3 dimerization is essential for PEAK3 phospho-tyrosine signaling. Overall, these findings support a tyrosine phosphorylation-dependent PEAK3 scaffolding activity, which implicates the TK PYK2.

**Figure 7:**
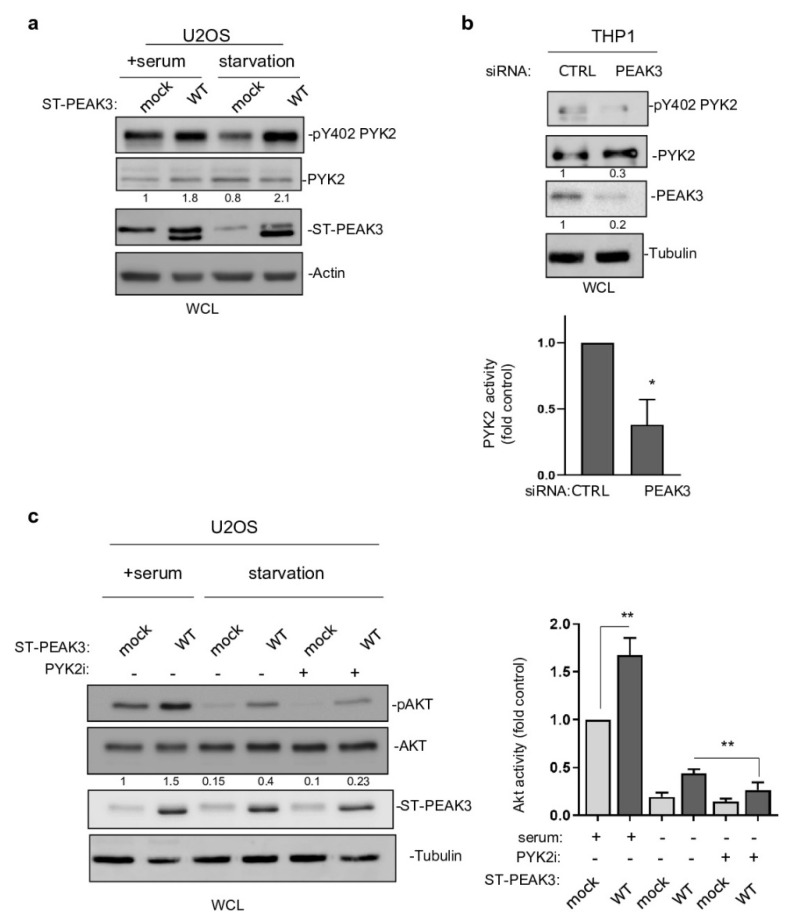
PYK2 activity mediates PEAK3–AKT signaling. (**a**) PYK2 activation by PEAK3 overexpressing U2OS cells that were serum-starved or not for >20 h. Western blots of the levels of PEAK3, PYK2, and pPYK2 (*n* = 2). (**b**) Regulation of PYK2 activity by endogenous PEAK3 in THP1 cells. PYK2 activity (pTyr402 PYK2 level) was measured in cells transfected with the indicated siRNAs. Quantification of PYK2 activity (mean ± SD; *n* = 3); * *p* < 0.05 (Student’s *t* test) (bottom). (**c**) PYK2 inhibition reduces PEAK3 activation of AKT in the absence of growth factors. U2OS cells that express or do not express PEAK3 were serum starved overnight or not, in the presence of PYK2 inhibitor (PYK2i) (1 μM) or vehicle (DMSO) as indicated. Western blots of the level of PEAK3, AKT, and pAKT (left) and relative quantification of AKT activity (mean ± SD; *n* = 3); ** *p* < 0.01 (Student’s *t* test) (right).

This PEAK3 activating mechanism on PYK2 was then confirmed in PEAK3 overexpressing U2OS cells (Figure 7a). Importantly, this molecular effect was further enhanced in serum-starved conditions (Figure 7a), also supporting a growth factor-independent mechanism of PEAK3 activation of PYK2. PEAK3 knockdown reduced PYK2 activity in THP1 cells (Figure 7b), supporting the existence of a similar endogenous PEAK3–PYK2 signaling in leukemic cells. Having demonstrated the existence of PEAK3–AKT and PEAK3–PYK2 signaling, we then asked whether PYK2 would mediate the growth-independent AKT activation by PEAK3. Acute pharmacological inhibition of PYK2 (PYK2i) in U2OS reduced the stimulating effect of PEAK3 on AKT 3-fold, while this inhibitor had no effect on AKT activity in control cells (Figure 7c). Overall, these findings demonstrate the fact that that PEAK3 activates PYK2 as well as the support role of PYK2 activity in PEAK3–AKT signaling.

## 3. Discussion

In this study, we show that the pseudo-kinase PEAK3 has an oncogenic function, as previously reported for PEAK1 and 2. PEAK3 overexpression enhanced cancer cell growth and migration, while its silencing reduced these pro-tumor effects. The PEAK3 expression profile suggested a restricted function in myeloid cells, such as granulocytes, and our results reveal a pro-tumoral function in AML. This finding was supported by the aberrant PEAK3 expression observed in AML patient samples and by the finding that endogenous PEAK3 is implicated in the regulation of THP1 leukemic cell features. As PEAK1/2 can mediate TK signaling induced by growth factor receptors [4,5], the absence of association between PEAK3 and the oncogenic receptor TK FLT3-TD in AML [28] is surprising. This suggests that PEAK3 may not be a critical component of the receptor TK signaling or that it displays redundant signaling functions. This hypothesis is corroborated by the finding that PEAK3 activates AKT signaling in the absence of growth factors. Moreover, the heterogeneous PEAK3 expression in AML patient samples suggests a PEAK3 function in a specific AML subtype that needs to be thoroughly characterized. The association between PEAK3 expression and DNTM3A [28] is consistent with this hypothesis.

This study also uncovers an essential role of the SHED module in PEAK3 biological activities. Specifically, PEAK3 dimerization was necessary for PEAK3 pro-tumoral activity, intracellular signaling, and localization to FAs. PEAK3 dimerization was also essential for interaction with important signaling proteins, uncovering a general scaffolding mechanism regulated by this dimeric domain to allow protein interactions. As this domain is conserved among PEAK family members, we might predict a similar function in PEAK1 and PEAK2 signaling that needs to be confirmed. Liu et al. reported a hetero-dimerization mechanism involved in PEAK1/2 signaling specificity [20]. We did not address whether this mechanism contributes also to PEAK3 signaling in this study. Nevertheless, the presence of another PEAK member in our PEAK3 interactomes suggests that heterodimers may contribute to PEAK3 signaling. However, as PEAK3 was the main PEAK member expressed in blood cells (Figure S1), PEAK3 signaling may occur primarily via PEAK3 homo-dimerization. Our imaging analysis revealed that nuclear PEAK3 localization is not affected by protein dimerization. This suggests that the SHED module does not regulate all PEAK3 functions, and that PEAK3 has an additional nuclear function, as described for PEAK2 [29], which remains to be characterized.

One important finding from our molecular study is the identification of PEAK3 as a novel upstream activator of AKT signaling, which plays an essential role in AML development [30]. This result corroborates a previous report showing that PEAK3 is a potential regulator of AKT signaling using a shRNA screening approach [31], although this observation was not further investigated. Our results also suggest that PEAK3 might define an important mechanism of AKT activation in the absence of growth factors. This mechanism could be essential for malignant cell survival and dissemination during disease progression. Our interactomic analysis also showed that PYK2 is an important mediator of this signaling cascade. This is consistent with FAK-like functions described in AML, where high FAK expression level is associated with enhanced blast migration, increased cellularity, and poor prognosis [32]. Mechanistically, our findings support a tyrosine phosphorylation-dependent PEAK3 signaling implicating the TK PYK2 (Figure S9). For instance, PEAK3 may act a scaffold to bring PYK2 and ASAP1 together for efficient ASAP1 tyrosine phosphorylation, enabling AKT signaling [33,34,35]. Moreover, our results suggest that PEAK3 activates PYK2 through a SHED-dependent mechanism, as previously reported for PEAK2 activation of CSK [8]. Therefore, we propose that, in addition to its scaffolding function, PEAK3 and PEAK2 use an allosteric mechanism to activate associated TKs for signaling. Since PYK2 is regulated by dimerization [26], its binding to PEAK3 might facilitate this activation mechanism. During the preparation of this manuscript, the Daly group reported a similar PEAK3 oncogenic function in breast epithelial cells and showed that PEAK3 tyrosine phosphorylation promotes GBR2 and ASAP1 binding [35], which is highly consistent with our proposed mechanism of PEAK3 oncogenic signaling.

## 4. Material and Methods

### 4.1. Antibodies and Constructs

The anti-PEAK3 antibody was generated against a bacterially produced recombinant His-TRX-PEAK3 fusion protein [36]. Briefly, the His-tagged protein was purified in a denaturing condition in the presence of 8M guanidine hydrochloride, followed by extensive dialysis against PBS before rabbit immunization [37]. Anti-PEAK3 antibodies were affinity-purified against the His-PEAK3 that was blotted on a PVDF membrane [38]. Anti-ERK1/2, anti-ERK1/2 pT202/Y204, anti-AKT, anti-AKT pS473, anti-pSer/pThr AKT pan-substrate, anti-GRB2, anti-ASAP1, anti-CRK II, anti-PYK2, anti-pPYK2 pTyr402, and anti-PYK2 pTyr881 antibodies was sourced from CST; the anti-ASAP1 used for immunoprecipitation assays was sourced from Bethyl A302; the anti-actin, anti-tubulin, and 4G10 antibody were gifts from N Morin (CRBM); the anti-MYC antibody 9E10 was sourced from Origene, the anti-Strep-tag antibody from Biorad, the anti-rabbit IgG-HRP and anti-mouse IgG-HRP antibodies from GE Healthcare, and the anti-HA antibody 12CA5 from Sigma. The PYK2 inhibitor PF-431396 (1 μM) was sourced from Selleck Biochem and the pan-PI3K inhibitor LY294002 (10 μM) from Sigma. 

The codon-optimized PEAK3 (GenBank NM198532) in the pDONR221 vector was obtained from DNASU (catalogue number HsCD00813413) and subcloned in the pBabe-puro GFP-N-term pCDNA-T0-FRT-Streptag [39] and pSBbi-PUR Sleeping Beauty-based expression vectors (Addgene #6023, a gift from E. Kowarz) [40]. The PEAK3 A436E mutant was generated with the QuickChange Site-Directed Mutagenesis Kit (Stratagene). The pCDNA3.1-V5-PTK2B (PYK2) plasmid was a gift from Kai Johnsson (Addgene plasmid # 127233). The siRNAs were: 5′TTCTCCGAACGTGTCACGTTT3′ (siCTRL), 5′GTATAGCAACCTTGGTCAGAT 3′ (siPEAK3 also named siPEAK3 #1), GCGGGGACGCCCUGUAUUA (siPEAK3 #2), and GGUCAGCGUCUCCAUGAUA (siPEAK3 #3) [35].

### 4.2. Phylogenetic Analyses and Data Mining

Nucleic and protein PEAK sequences were retrieved from the NCBI annotated non-redundant database (http://www.ncbi.nlm.nih.gov accessed on 15 September 2020) using NCBI PHI-BLAST, BLAST, and the Annotation search tools available in the Geneious 11.1.5 software package (Biomatters, http://www.geneious.com accessed on 15 September 2020). Accession numbers are listed in Table S1. Protein sequences were aligned using MAAFT v7.450 [41]. Protein multiple sequence alignments (MSA) were processed using BMGE (Block Mapping and Gathering with Entropy) [42] and a cut-off value = 0.6. Phylogenetic trees were estimated using PhyML [43], using the General Time Reversible model with invariant and gamma decorations, and MrBayes [44] with two independent runs of four Markov chains for 1,000,000 generation samples for every 200 generations. Sample trees from the first 100,000 generations were discarded as “burn-in”. At the end of the two runs, the split frequency standard deviations were ~1.5 × 10^−3^, and the effective sample sizes were >2600. The non-synonymous to silent substitution ratios (ω = dN/dS) were calculated using PAML 4.4 [45]. Nucleic MSAs were based on protein alignments. The “one-ratio” (i.e., a single ω ratio for the entire tree) and the two “two-ratio” models (i.e., distinct ω values for two or three PEAK branches) were compared using the likelihood-ratio test. Transcriptomic signatures and data mining from two PEAK3 signatures were generated from the transcriptomes of the acute myeloid leukemia (AML) patient samples, high versus low PEAK3 expression from TCGA transcriptomic data (gepia.cancer-pku.cn), and the BEATAML database (GSE6891) and correlated with well-characterized oncogenic somatic mutations.

### 4.3. Cell Cultures and Transfections, Cell Growth and Migration

Adherent cell lines (ACTT; Rockville, MD) were cultured at 37 °C and 5% CO_2_ in a humidified incubator in Dulbecco’s Modified Eagle’s Medium GlutaMAX. Blood cell-derived cell lines were grown in RPMI (Thermo Fisher Scientific, Waltham, MA, USA) supplemented with 10% fetal calf serum (FCS), 100 U/mL of penicillin, and 100 µg/mL of streptomycin. Cell treatment with pharmacological inhibitors was performed as in [11]. Cell transfections were performed as described in [46]. U2OS, HeLa, and THP1 cell lines that stably express GFP- and Strep-tagged (ST)-PEAK3 were obtained by transfection of the pBABE-GFP-PEAK3 and pSBbi-PUR-ST-PEAK3 constructs, respectively, with TurboFect™ (TurboFect™, followed by puromycin selection. HEK293T cells were transiently transfected with jetPEI^®^ (Polyplus-transfection) according to the manufacturer’s instructions (Polyplus Illkirch, France). For siRNA transfection, 2 × 10^5^ cells were seeded in 6-well plates and transfected with 20 nmol of siRNAs and 9 µL of Lipofectamine^®^ RNAi Max for 3 days before use, according to the manufacturer’s protocol (Thermo Fisher Scientific, Waltham, MA, USA). Cell growth was measured using Sulforhodamide B staining (Sigma-Aldrich, St. Louis, MO, USA), and invasion was assessed using Boyden chambers coated with Matrigel (1 mg/mL) [11]. Cell transmigration was monitored as described in [47]. 

### 4.4. Human Samples

Primary AML samples from patients (with annotated clinical and biological characteristics, treatments, and outcome data) were obtained from the Biobank of the Hematology Clinical Department, Toulouse University Hospital, France, (HIMIP collection, BB-0033-00060; INSERM-U1037) headed by Prof C. Récher. The recording in the database of clinical and biological data has been declared to the CNIL (Comité National Informatique et Libertés; the French Committee for the protection of personal data). Samples are routinely characterized by morphologic, immunophenotypic, cytogenetic, and molecular (FLT3-ITD, NPM1, CEBPA, DNMT3A, KIT, IDH1, and IDH2 mutations) analyses. 

### 4.5. Biochemistry and Proteomics

Immunoprecipitation and immunoblotting were performed as described in [46]. Briefly, cells were lysed at 4 °C with lysis buffer (20 mM Hepes pH 7.5, 150 mM NaCl, 0.5% Triton X-100, 6 mM β-octylglucoside, 10 µg/mL aprotinin, 20 µM leupeptin, 1 mM NaF, 1 mM DTT, and 100 µM sodium orthovanadate). Then, 20–50 µg of whole cell lysates was loaded on SDS-PAGE gels and, after electrophoretic separation, transferred onto Immobilon membranes (Millipore, Burlington, MA, USA). After incubation with the relevant antibodies, detection was performed with the ECL System (Amersham Biosciences, Amersham, UK). Original blots can be found in Figure S10.

The Proteome Profiler Human Phospho-Kinase Array Kit was purchased from R&D Systems. THP1 cells were lysed, and 300 μg of protein lysates was used for western blotting according to the manufacturer’s protocol. Signals were quantified with the Amersham Imager 600 (GE Healthcare, Chicago, IL, USA) from two independent biological replicates. 

Interactomic analyses were performed as described in [48]. Cell lysates were incubated with streptavidin beads (Thermo Fisher Scientific, Waltham, MA, USA) overnight and washed three times with a lysis buffer and once with a washing buffer (50 mM Tris pH 7.5 and 50 mM NaCl). PEAK3 complexes were eluted with 2.5 mM biotin in 50 mM Tris pH 8 and 0.5 mM EDTA and processed for mass spectrometry (MS) analysis. Peptides obtained after digestion were analyzed using a Qexactive-HFX system coupled with a RSLC-U3000 nano HPLC. Samples were desalted and pre-concentrated online on a Pepmap^®^ precolumn (0.3 mm × 10 mm). A gradient consisting of 6–25% B for 100 min, 25–40% B for 20 min, and 40–90% B for 2 min (A = 0.1% formic acid; B = 0.1% formic acid in 80% acetonitrile) at 300 nl/min was used to elute peptides from the capillary reverse-phase column (0.075 mm × 250 mm; Pepmap^®^, Thermo Fisher Scientific) fitted with a stainless steel emitter (Thermo Scientific). Spectra were acquired with the instrument operating in data-dependent acquisition mode throughout the HPLC gradient. MS scans were acquired with resolution set at 60,000. Up to twelve of the most intense ions per cycle were fragmented and analyzed using a resolution of 300,000. Peptide fragmentation was performed using nitrogen gas on the most abundant and at least doubly-charged ions detected in the initial MS scan and a dynamic exclusion time of 20 s. Analysis was performed using the MaxQuant software (version 1.5.5.1, Max Planck Institute of Biochemistry, Munich, Germany). All MS/MS spectra were searched using Andromeda against a decoy database consisting of a combination of the *Homo sapiens* reference proteome (release 4 February 2019 www.uniprot.org) and of 250 classical contaminants, containing forward and reverse entities. A maximum of two missed cleavages was allowed. The search was performed with oxidation (M) and acetyl (protein N-term) as variable modifications and carbamidomethyl (C) as a fixed modification. FDR was set at 0.01 for peptides and proteins, and the minimum peptide length was 7. The *t-*test and graphical representation were performed with Perseus (v 1.6.10.43, Max Planck Institute of Biochemistry, Munich, Germany) and standard parameters. 

### 4.6. Cell Imaging

Cells seeded on microscope coverslips were fixed with 4% paraformaldehyde in a Triton-X100-BRB80 buffer (0.5% Triton-X100, 80 mM PIPES pH 6.8, 1 mM MgCl_2_, and 1 mM EGTA) containing 5% FCS at 37 °C for 20 min. Cells were incubated with the indicated primary antibodies and the corresponding secondary antibodies labelled with Alexa Fluor dyes (488 or 594 nm) and DAPI diluted in PBS buffer. After mounting with a ProLong Antidafe Mountant (Thermo Fisher), cells were observed with a DMR A microscope and a PL APO 63× oil objective (1.32 NA). Images were captured using a piezzo stepper (E662 LVPTZ amplifier; Servo) and a cooled CCD Micromax camera (1300 × 1030 pixels, RS; Princeton Instruments Inc. Trenton, NJ, USA) driven by MetaMorph (v 4.5; Molecular Devices San Jose, CA, USA). Images were processed with ImageJ (https://imagej.nih.gov/ij/ accessed on 6 June 2021).

### 4.7. Statistical Analysis 

All statistical analyses were performed on data from at least three independent experiments with GraphPad Prism (Version 9, GraphPad Software, San Diego, CA, USA). Data are presented as the mean ± standard deviation (SD). When distribution was normal (assessed with the Shapiro–Wilk test), the two-tailed *t* test was used for between-group comparisons. Otherwise, the Mann–Whitney test was used.

## 5. Conclusions

Our study provides a unifying model on how PEAK pseudo-kinases regulate oncogenic signaling. GRB2 and possibly CRK proteins are common PEAK1-3 binders [8,20,21], through a conserved proline-rich sequence at their N-terminus. Moreover, our interactomic analyses identified a unique set of signaling proteins recruited by PEAK3 that were not detected in previous PEAK1/2 interactomes [8,20]. Altogether, these findings support the existence of a common SHED-dependent mechanism of PEAK1-3 oncogenic activity mediated through the recruitment of a unique set of signaling proteins to induce specific oncogenic functions. Lopez et al. found that the conserved DFG motif in the PEAK3 pseudo-kinase domain is implicated in PEAK3 dimerization [21]. This raises an interesting idea that, besides the SHED module, the pseudo-kinase domain might also participate in PEAK3 signaling. Consistent with this idea, recent molecular studies have shown that, despite the inaccessible ATP site found in crystallography, pseudo-kinases display higher kinase flexibility than previously expected to enable signaling [2,49,50]. Therefore, small molecules that affect pseudo-kinase dynamics may inhibit their function [49,50]. Whether PEAK1-3 signaling is regulated by a similar mechanism deserves investigation. In this case, these pseudo-kinases could represent attractive therapeutic targets in human cancers, including leukemia.

## Data Availability

All the datasets in this study are presented within the article and as supplementary information. Mass spectrometry proteomics data have been deposited to the ProteomeXchange Consortium with the dataset identifier PXD02941.

## Supplementary Materials

The following are available online at https://www.mdpi.com/article/10.3390/cancers13246344/s1: Figure S1: PEAK1-3 mRNA distribution in human tissues, Figure S2: Characterization of home-made anti-PEAK3 antibody, Figure S3: PEAK3 transcript level in AML patient samples, Figure S4: PEAK3 signaling in HeLa cells, Figure S5: PEAK3 silencing by siRNA PEAK3 #2 and #3 reduces THP1 cell growth and AKT signaling, Figure S6: PEAK3 cellular distribution, Figure S7: PEAK3–AKT signaling, Figure S8: SHED-dependent PEAK3 phospho-tyrosine signaling, Figure S9: A model on PEAK3 oncogenic signaling, Figure S10: Original Western-blot figures, Table S1: Accession numbers of the PEAK pseudo-kinases used in this study, Table S2: Characteristics of AML patient samples, and Table S3: Main hits recovered from the PEAK3 interactomic analyses in U2OS, HeLa and THP1 cells.
