# Supplementary material for: SHED-Dependent Oncogenic Signaling of the PEAK3 Pseudo-Kinase
Source: Cancers (Basel). 2021 Dec 17;13(24):6344. doi: 10.3390/cancers13246344 (PMC8699254; doi:10.3390/cancers13246344)
Supplement: Supplementary file 1 [file cancers-13-06344-s001.zip › supplementary information/Figure S10.pdf]

Figure 2a

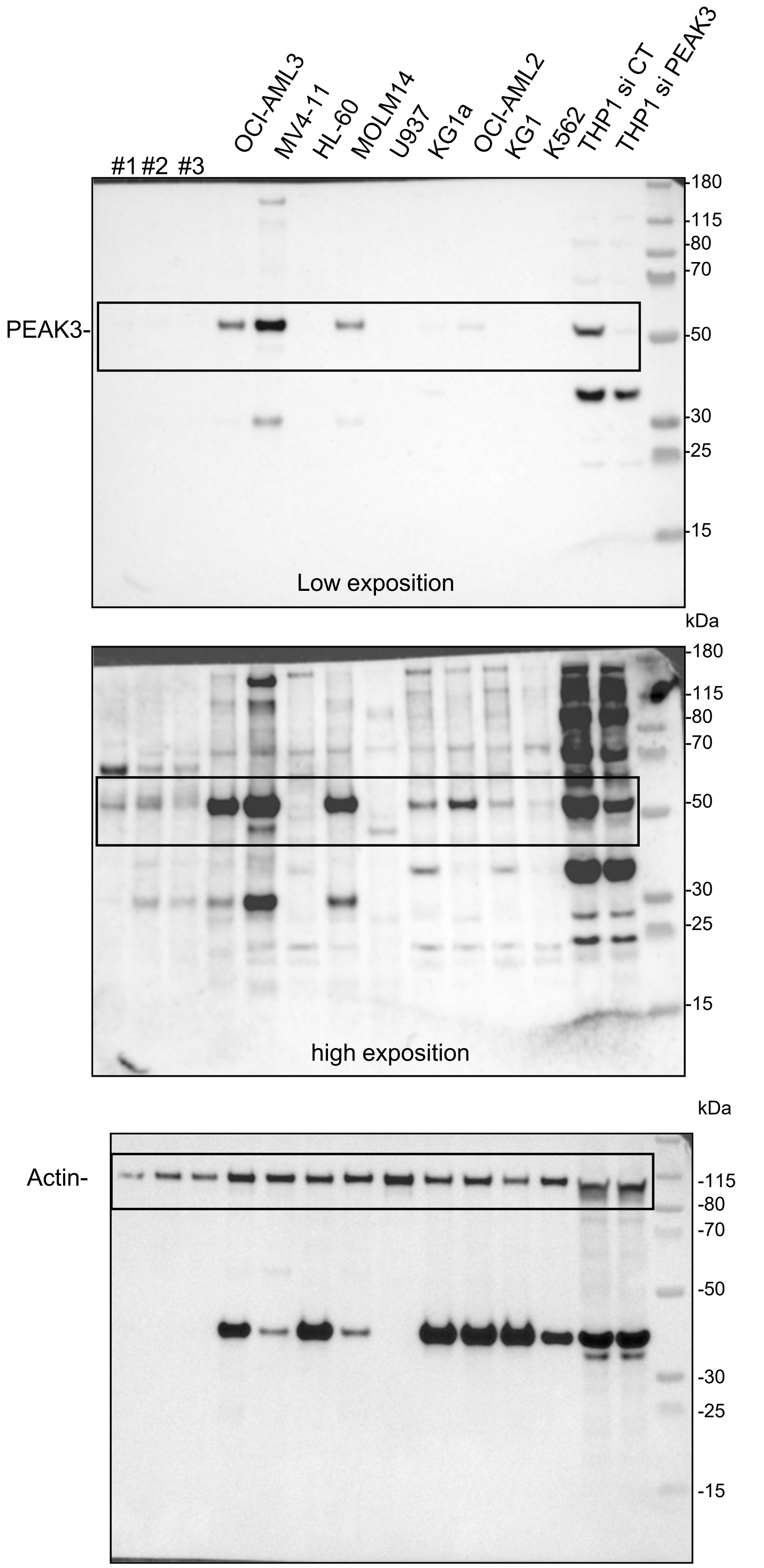
#1 #2 #3
OCI-AML3
MV4-11
HL-60
MOLM14
U937
KG1a
OCI-AML2
KG1
K562
THP1 si CT
THP1 si PEAK3
PEAK3-
180
115
80
70
50
30
25
15
Low exposition
kDa
high exposition
Actin-

Figure 2b

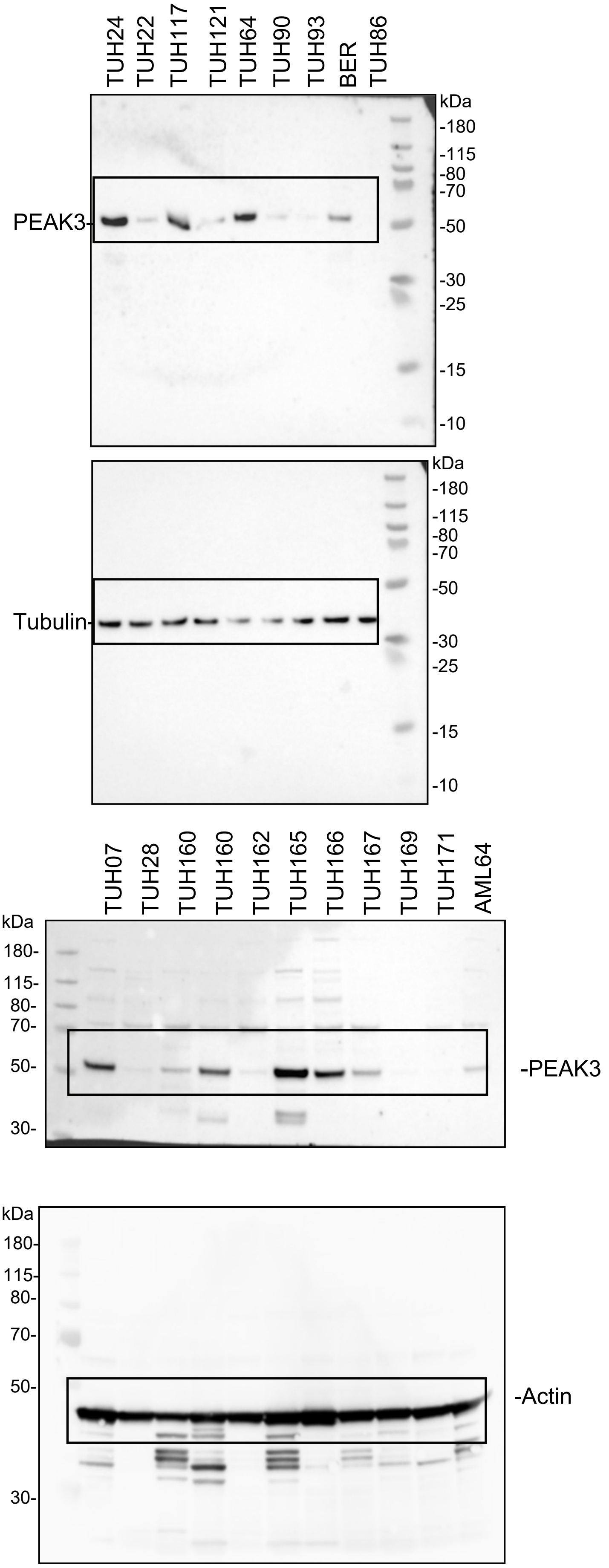

TUH24
TUH22
TUH117
TUH121
TUH64
TUH90
TUH93
BER
TUH86
kDa
-180
-115
-80
-70
-50
-30
-25
-15
-10
PEAK3
kDa
-180
-115
-80
-70
-50
-30
-25
-15
-10
Tubulin
TUH07
TUH28
TUH160
TUH160
TUH162
TUH165
TUH166
TUH167
TUH169
TUH171
AML64
kDa
180-
115-
80-
70-
50-
30-
-PEAK3
kDa
180-
115-
80-
70-
50-
30-
-Actin

Figure 3a

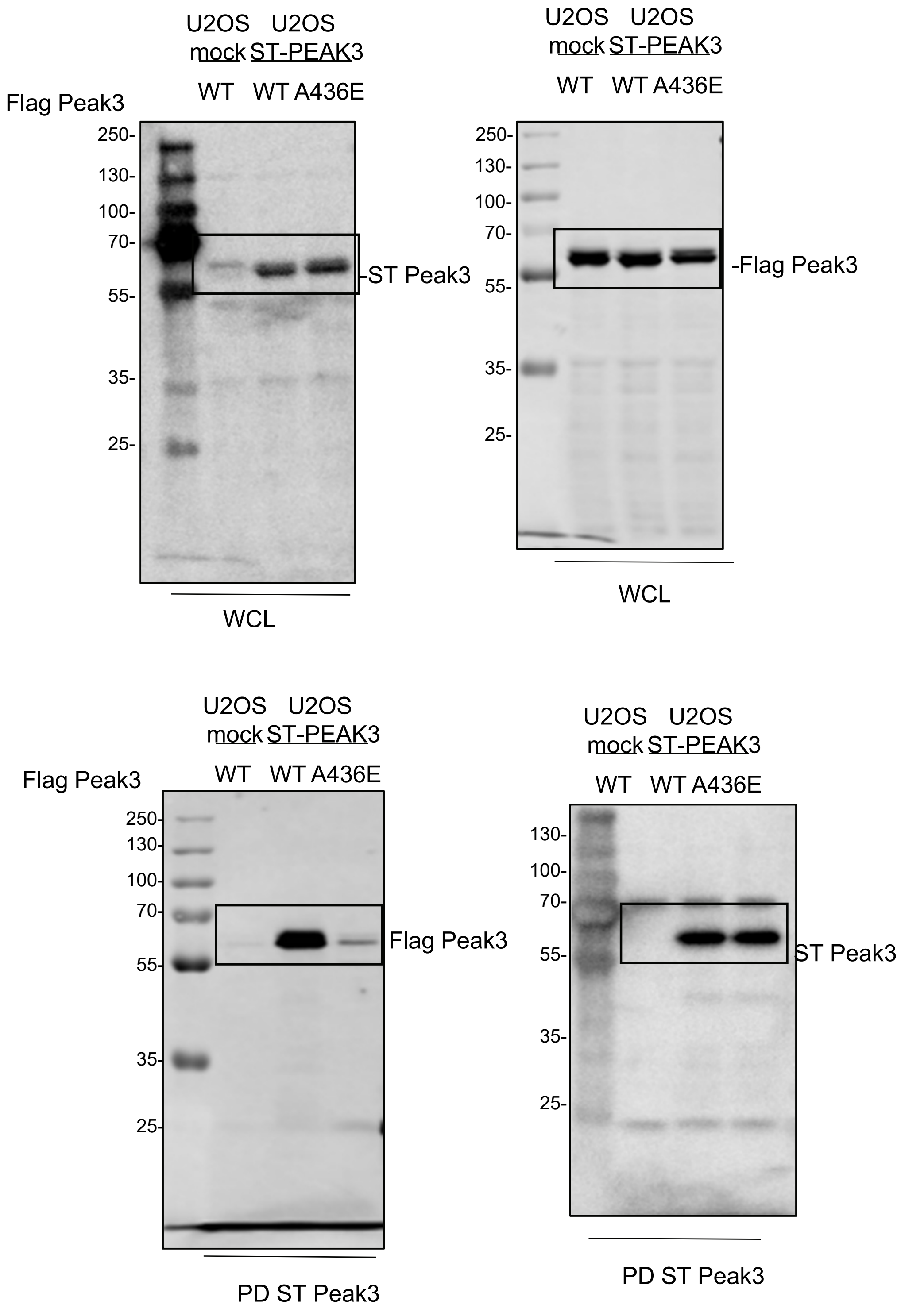
U2OS
U2OS
mock
ST-PEAK3
WT
WT
A436E
Flag Peak3
250-
130-
100-
70-
55-
35-
25-
-ST Peak3
WCL
-Flag Peak3
WCL
Flag Peak3
PD ST Peak3
ST Peak3
PD ST Peak3

Figure 3c and d

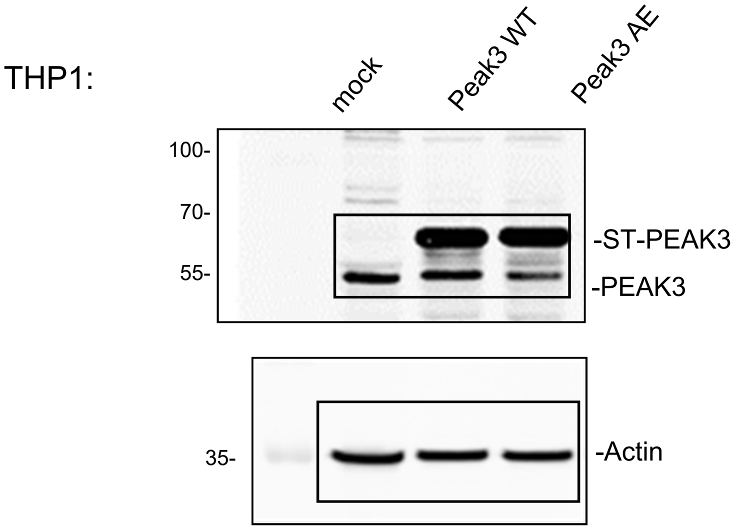

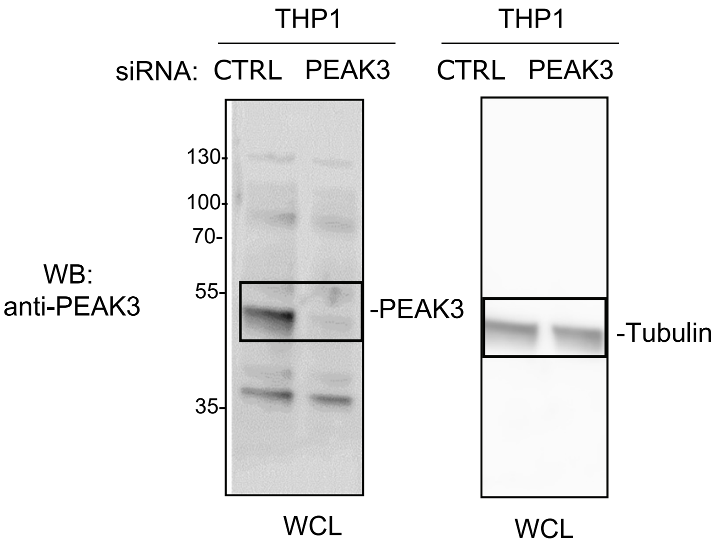

Figure 4a

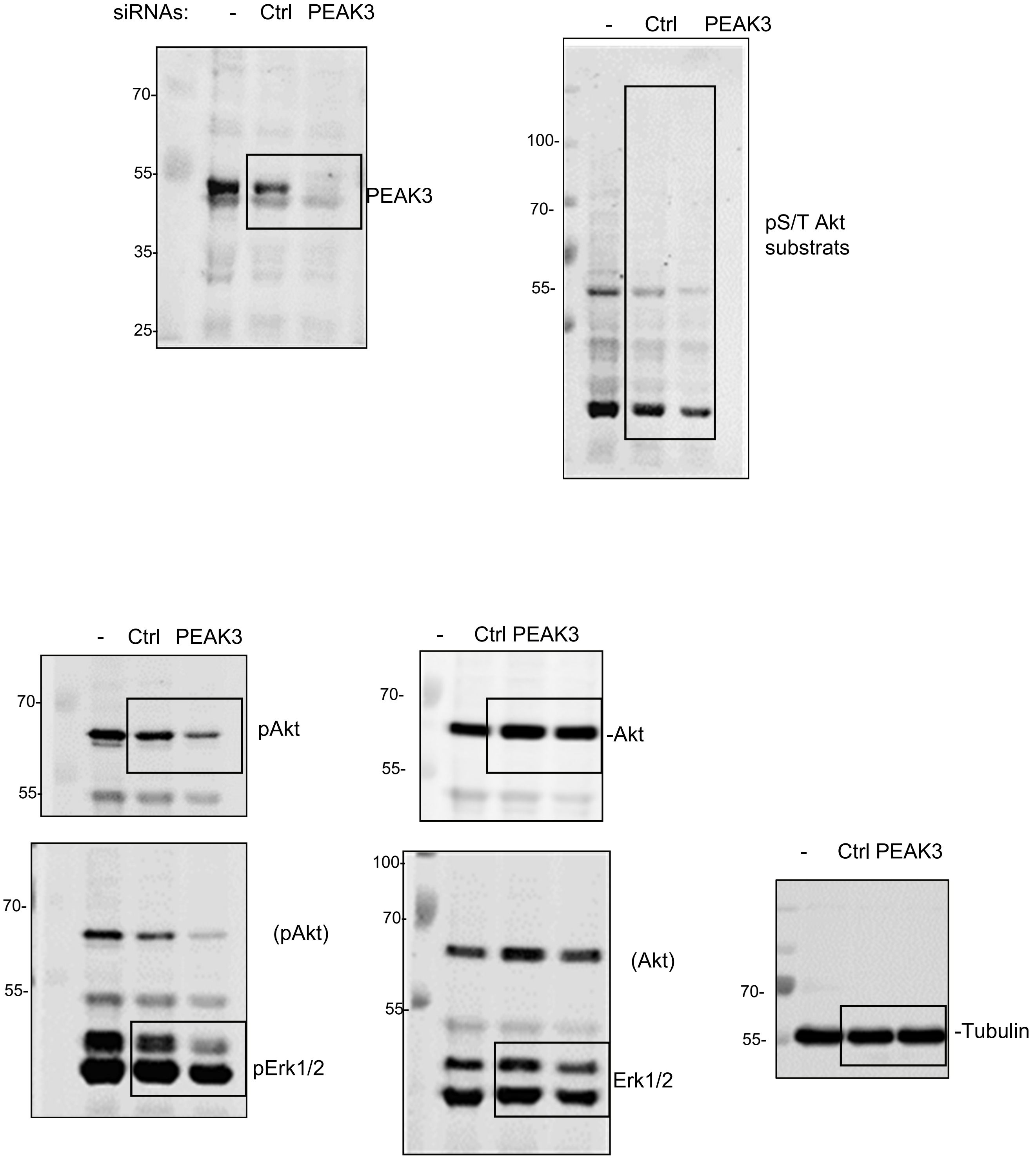

siRNAs: - Ctrl PEAK3
70-
55-
35-
25-
PEAK3
- Ctrl PEAK3
100-
70-
55-
pS/T Akt substrats
- Ctrl PEAK3
70-
55-
pAkt
- Ctrl PEAK3
70-
55-
-Akt
70-
55-
(pAkt)
pErk1/2
100-
70-
55-
(Akt)
Erk1/2
- Ctrl PEAK3
70-
55-
-Tubulin

Figure 4b

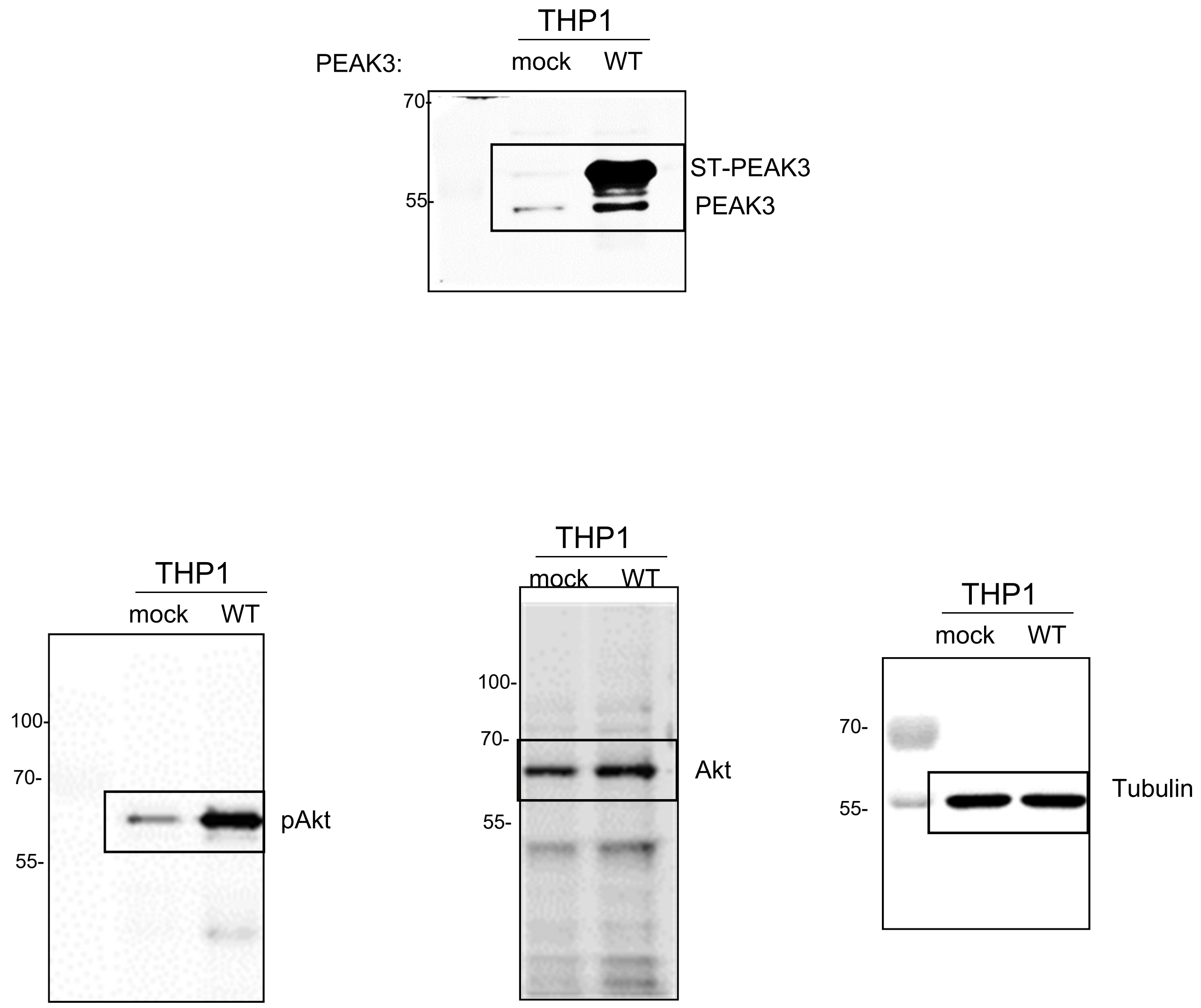
THP1
PEAK3:
mock
WT
70-
55-
ST-PEAK3
PEAK3
THP1
mock
WT
100-
70-
55-
pAkt
THP1
mock
WT
100-
70-
55-
Akt
THP1
mock
WT
70-
55-
Tubulin

Figure 4c

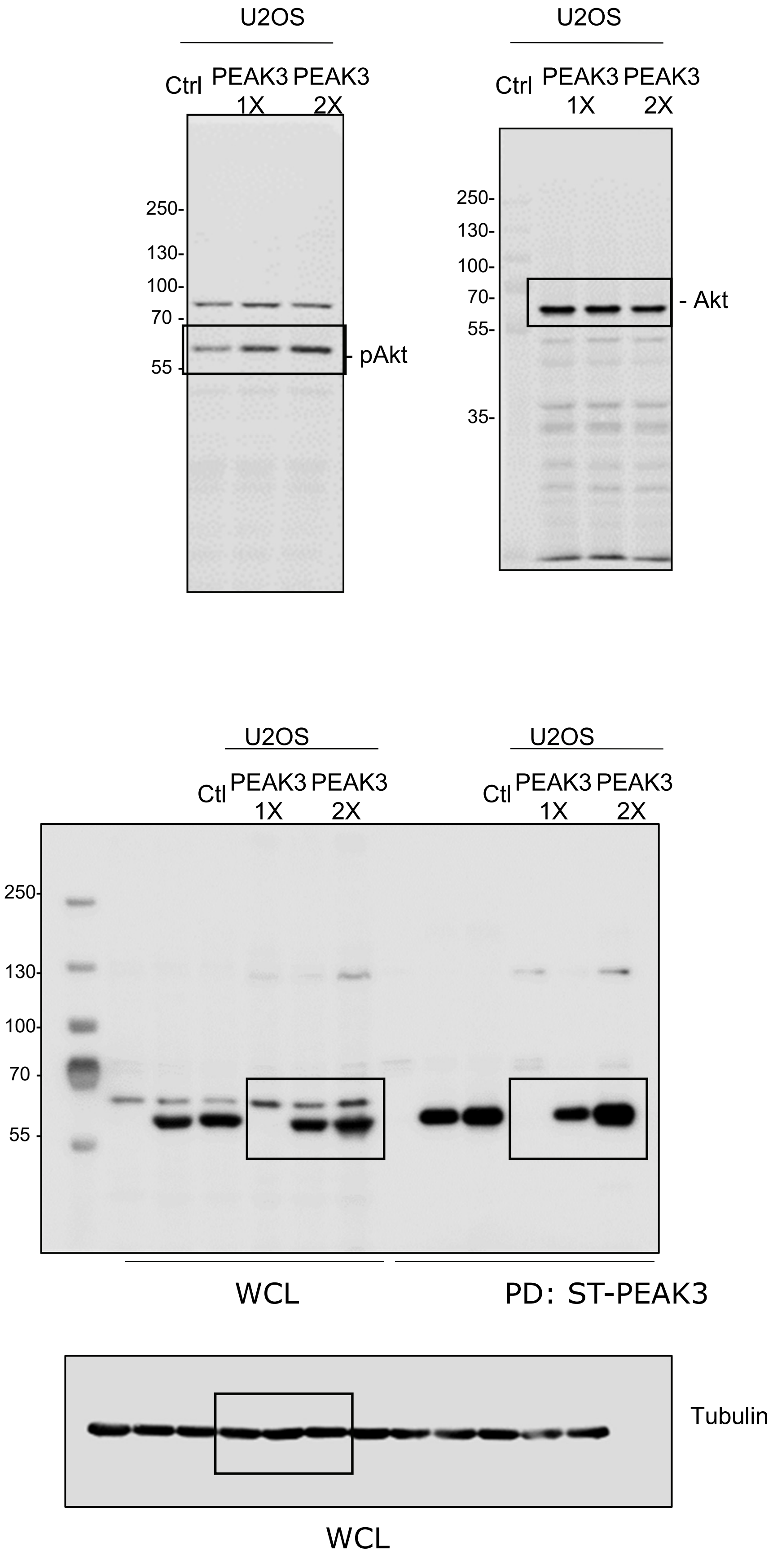

U2OS
Ctrl
PEAK3 1X
PEAK3 2X
250-
130-
100-
70 -
55 -
- pAkt
U2OS
Ctrl
PEAK3 1X
PEAK3 2X
250-
130-
100-
70-
55-
35-
- Akt
U2OS
Ctl
PEAK3 1X
PEAK3 2X
U2OS
Ctl
PEAK3 1X
PEAK3 2X
250
130
100
70
55
WCL
PD: ST-PEAK3
Tubulin
WCL

Figure 4c

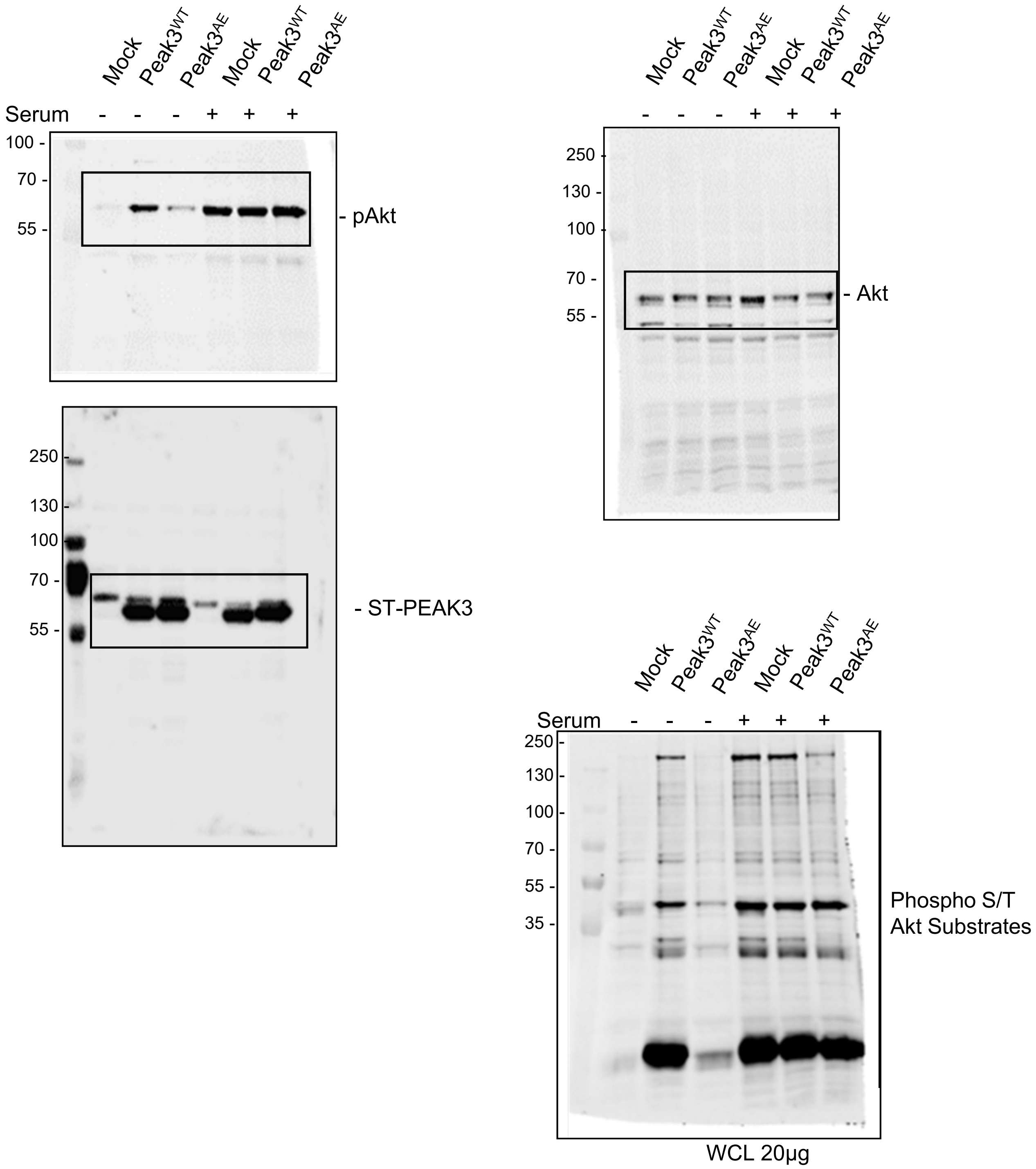

Mock
Peak3^{WT}
Peak3^{AE}
Mock
Peak3^{WT}
Peak3^{AE}
Serum - - - + + +
100 -
70 -
55 -
- pAkt
250
130
100
70 -
55 -
- ST-PEAK3
Mock
Peak3^{WT}
Peak3^{AE}
Mock
Peak3^{WT}
Peak3^{AE}
- - - + + +
250
130 -
100
70 -
55 -
- Akt
Mock
Peak3^{WT}
Peak3^{AE}
Mock
Peak3^{WT}
Peak3^{AE}
Serum - - - + + +
250
130
100
70 -
55 -
35 -
Phospho S/T
Akt Substrates
WCL 20µg

Figure 5b

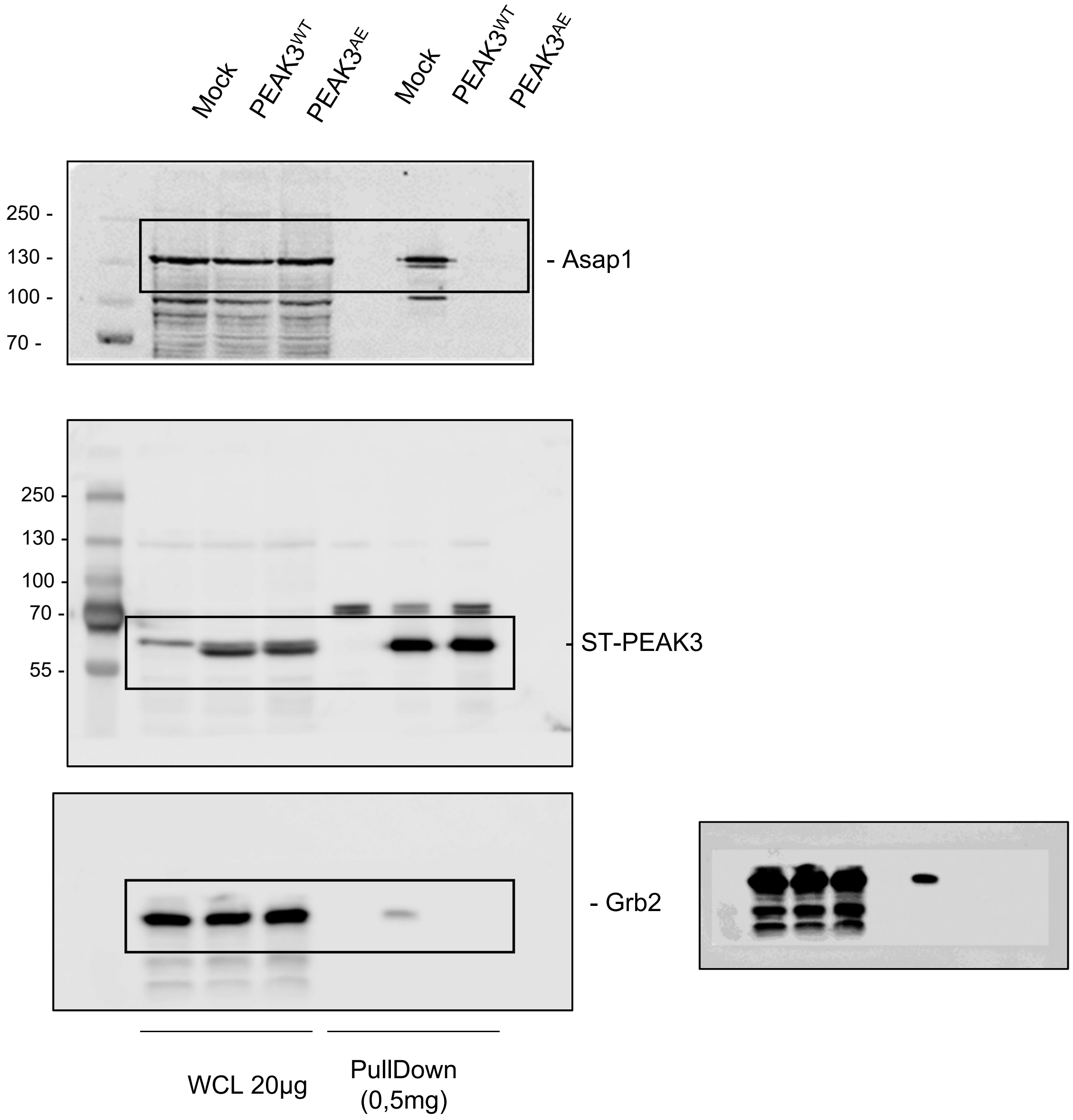

Mock
PEAK3WT
PEAK3AE
Mock
PEAK3WT
PEAK3AE
250 -
130 -
100 -
70 -
- Asap1
250
130
100
70
55
- ST-PEAK3
- Grb2
WCL 20µg
PullDown (0,5mg)

Figure 5c

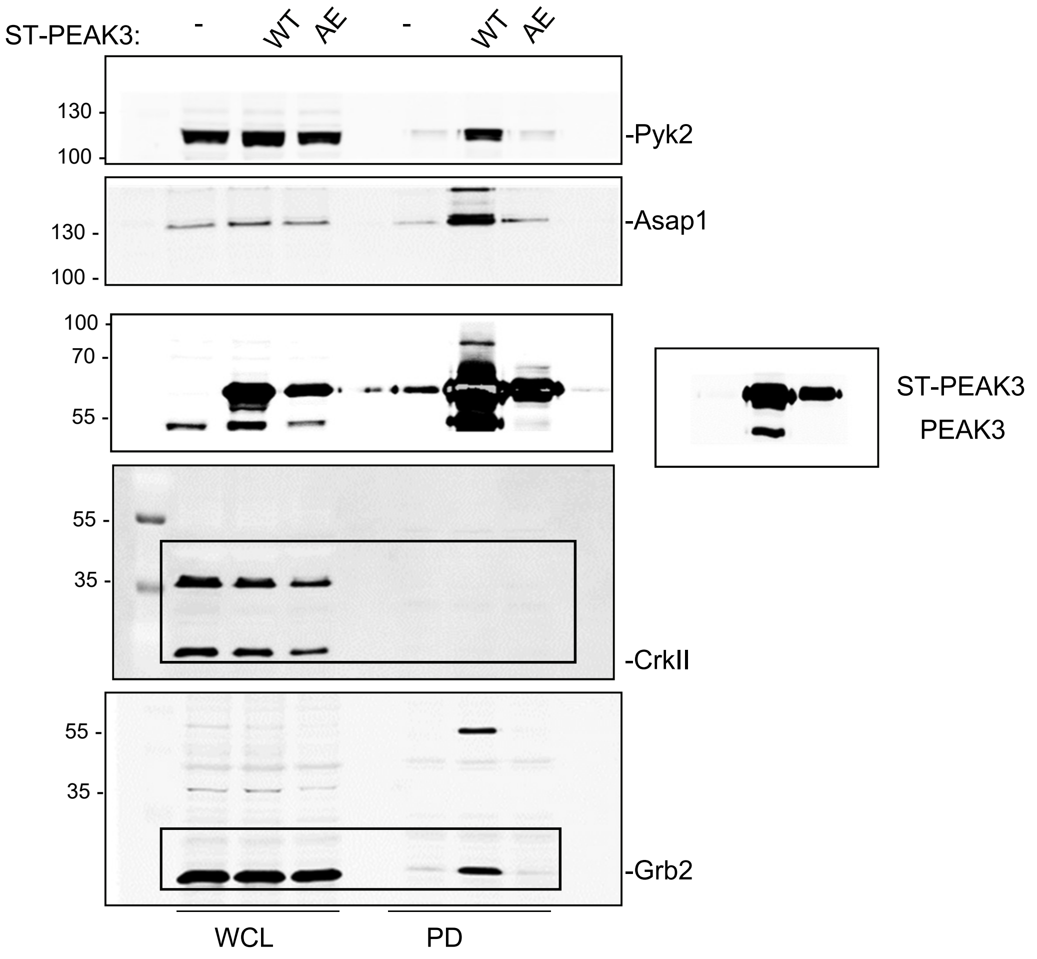

ST-PEAK3:
-
WT
AE
-
WT
AE
130
100
-Pyk2
130
100
-Asap1
100
70
55
ST-PEAK3
PEAK3
55
35
-CrkII
55
35
-Grb2
WCL
PD

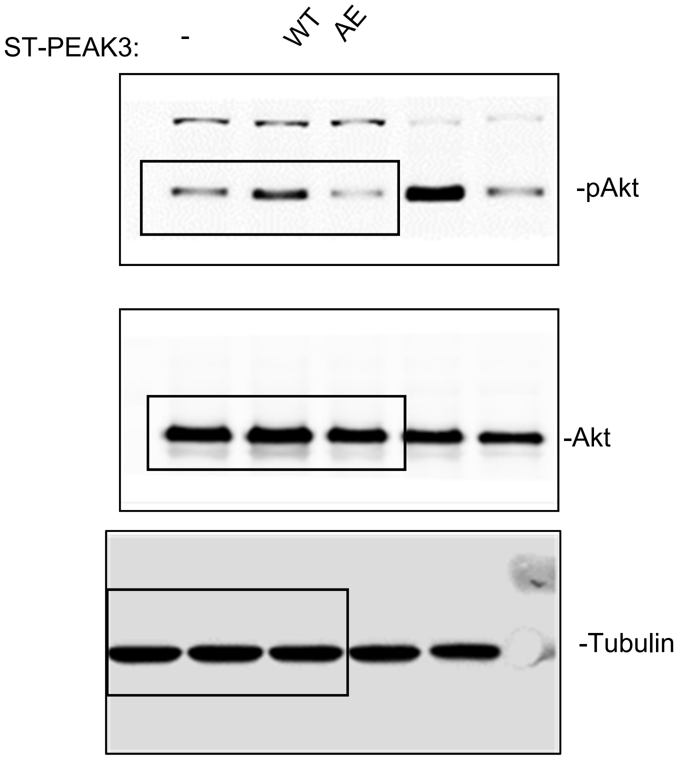

ST-PEAK3:
-
WT
AE
-pAkt
-Akt
-Tubulin

Figure 6a

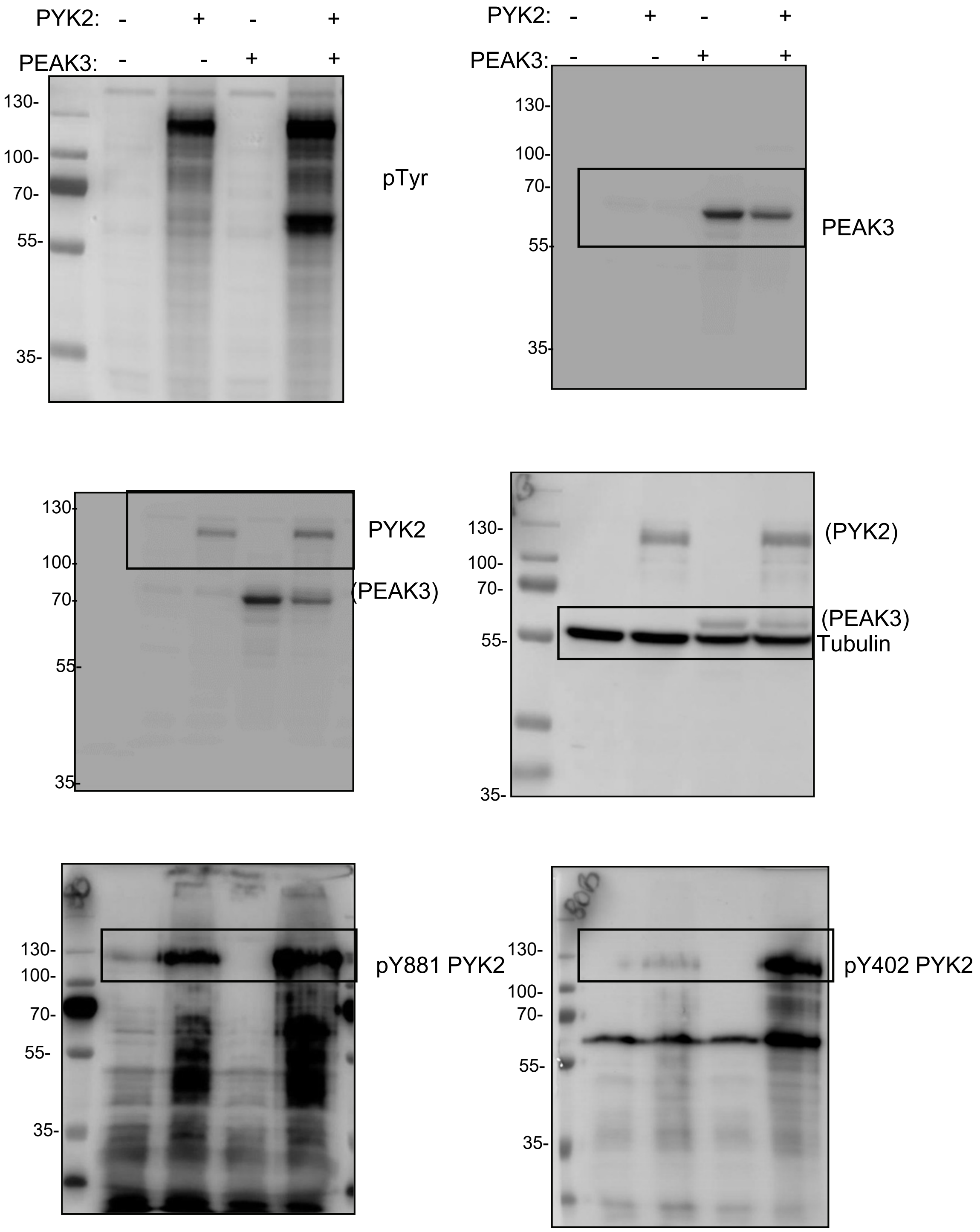
PYK2: - + - +
PEAK3: - - + +
130-
100-
70-
55-
35-
pTyr
PYK2: - + - +
PEAK3: - - + +
PEAK3
PYK2
(PEAK3)
(PYK2)
(PEAK3)
Tubulin
pY881 PYK2
pY402 PYK2

Figure 6b

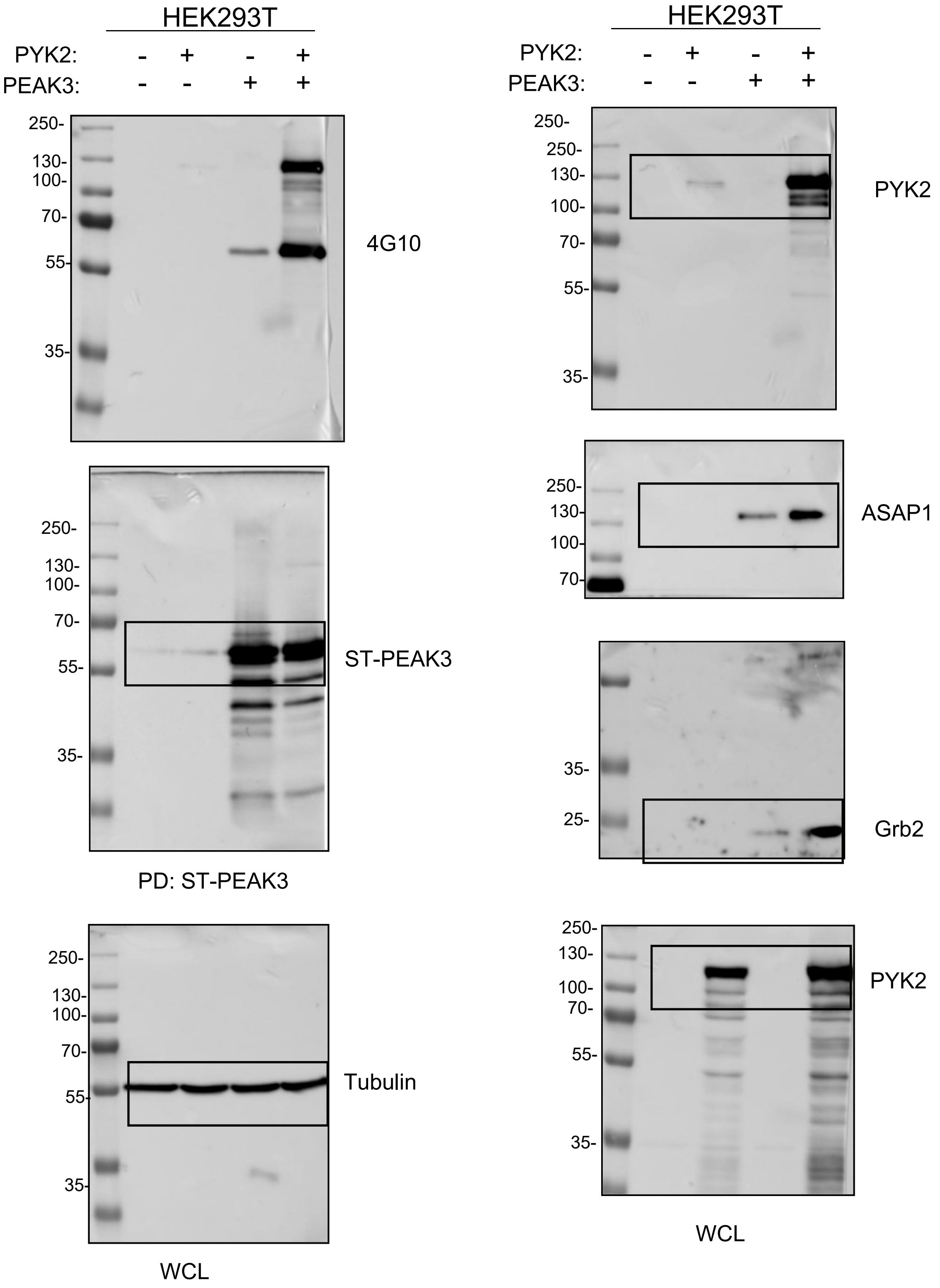
HEK293T
PYK2: - + - +
PEAK3: - - + +
250-
130-
100-
70-
55-
35-
4G10
ST-PEAK3
PD: ST-PEAK3
Tubulin
WCL
PYK2
ASAP1
25-
Grb2

Figure 6c

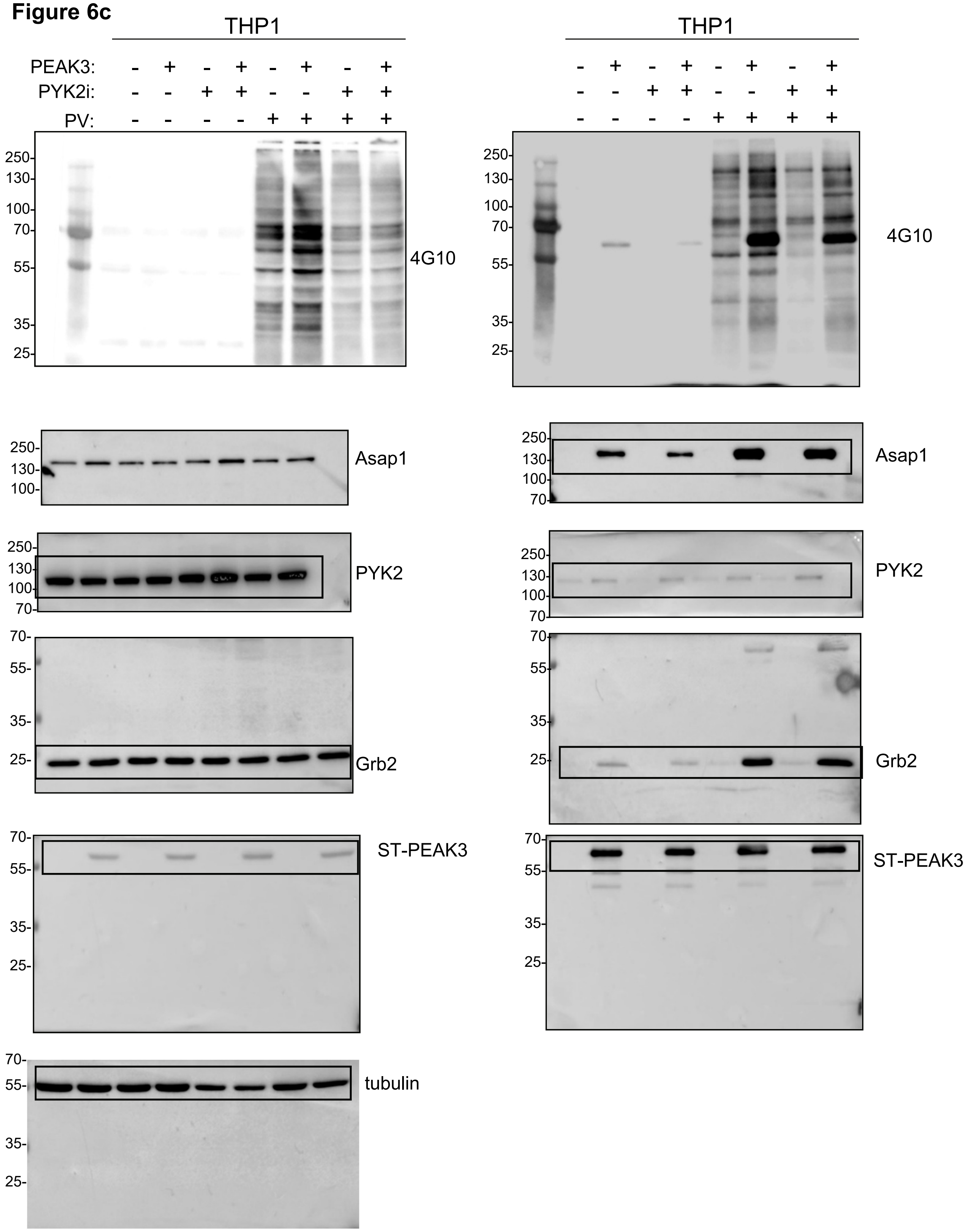
THP1
PEAK3: - + - + - + - +
PYK2i: - - + + - - + +
PV: - - - - + + + +
250-
130-
100-
70-
55-
35-
25-
4G10
Asap1
PYK2
Grb2
ST-PEAK3
tubulin
THP1
- + - + - + - +
- - + + - - + +
- - - - + + + +

Figure 6e

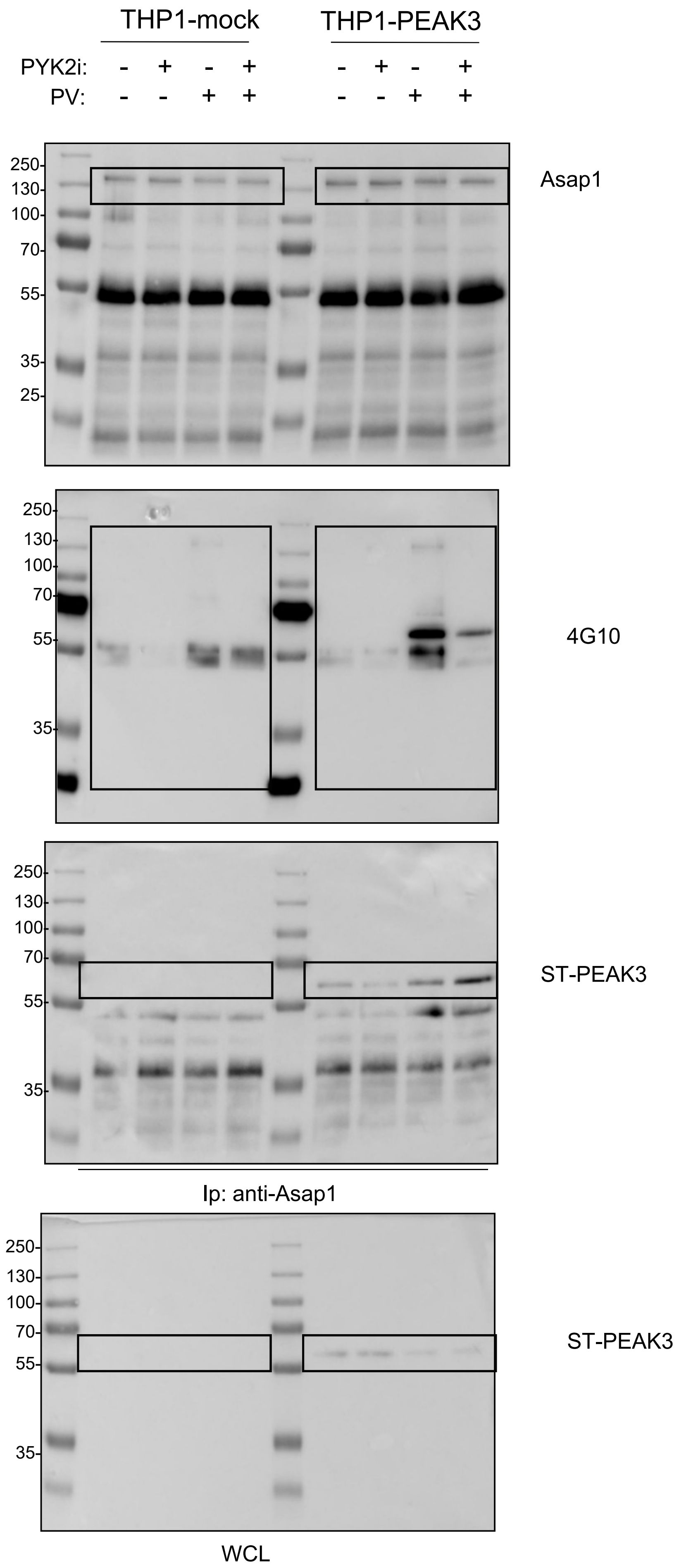
THP1-mock
THP1-PEAK3
PYK2i: - + - + - + - +
PV: - - + + - - + +
250-
130-
100-
70-
55-
35-
25-
Asap1
4G10
ST-PEAK3
Ip: anti-Asap1
ST-PEAK3
WCL

Figure 7a

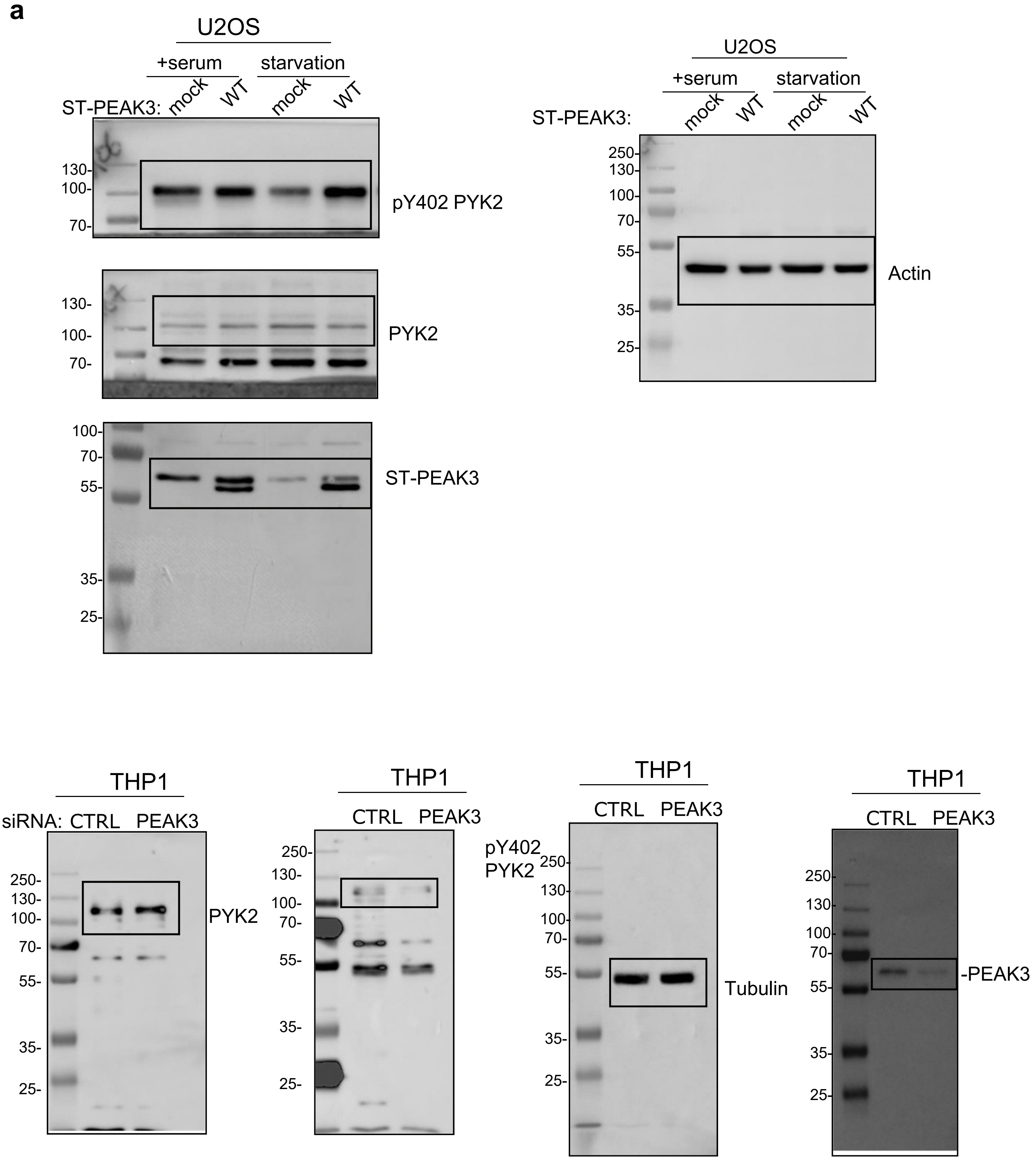
a
U2OS
+serum
starvation
ST-PEAK3:
mock
WT
mock
WT
pY402 PYK2
PYK2
ST-PEAK3
Actin
THP1
siRNA:
CTRL
PEAK3
pY402 PYK2
Tubulin
-PEAK3
250-
130-
100-
70-
55-
35-
25-

Figure 7b

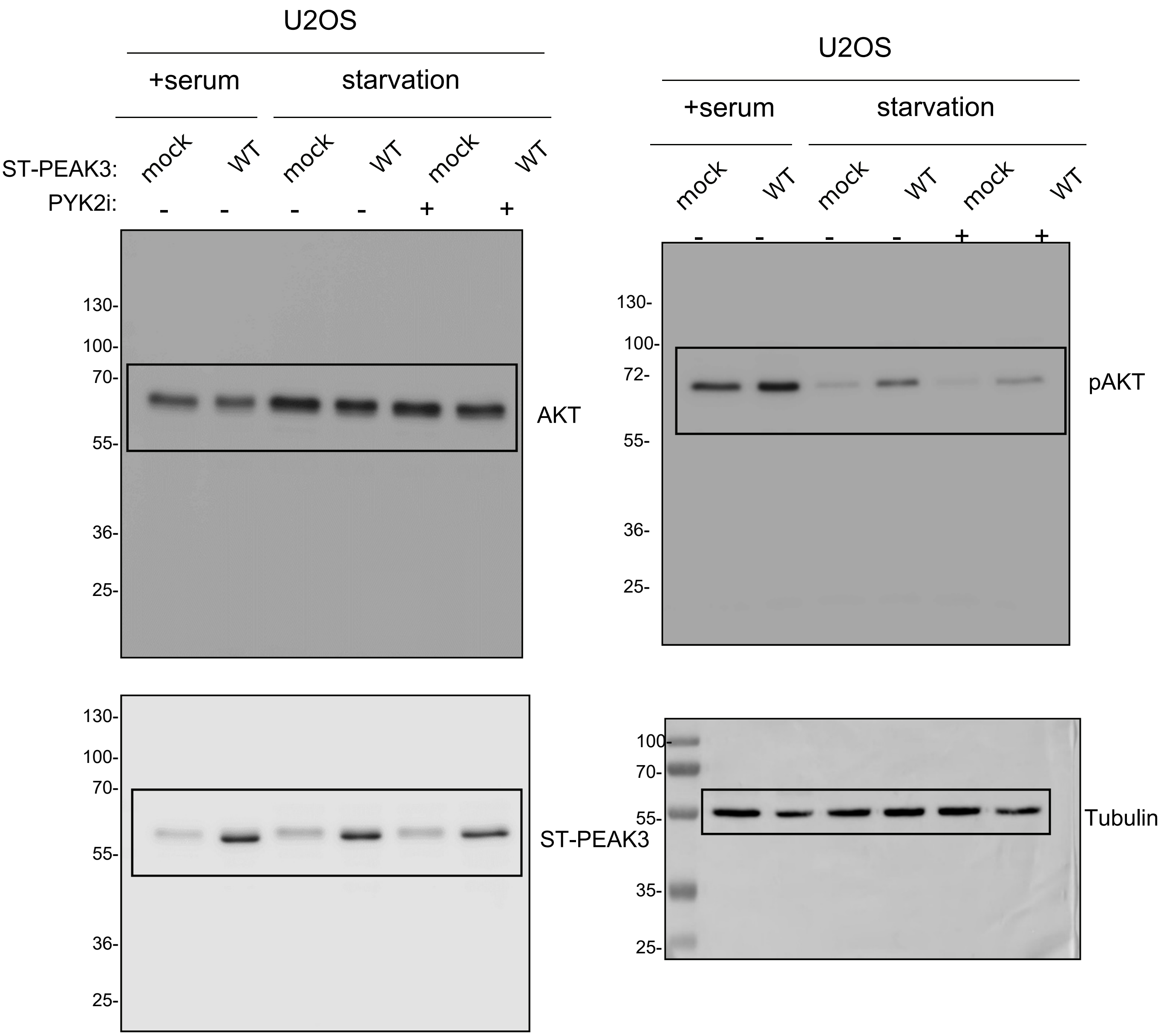

U2OS
+serum
starvation
ST-PEAK3:
mock
WT
mock
WT
mock
WT
PYK2i:
- - - - + +
130-
100-
70-
55-
36-
25-
AKT
U2OS
+serum
starvation
mock
WT
mock
WT
mock
WT
- - - - + +
130-
100-
72-
55-
36-
25-
pAKT
130-
100-
70-
55-
36-
25-
ST-PEAK3
100-
70-
55-
35-
25-
Tubulin

Figure S2

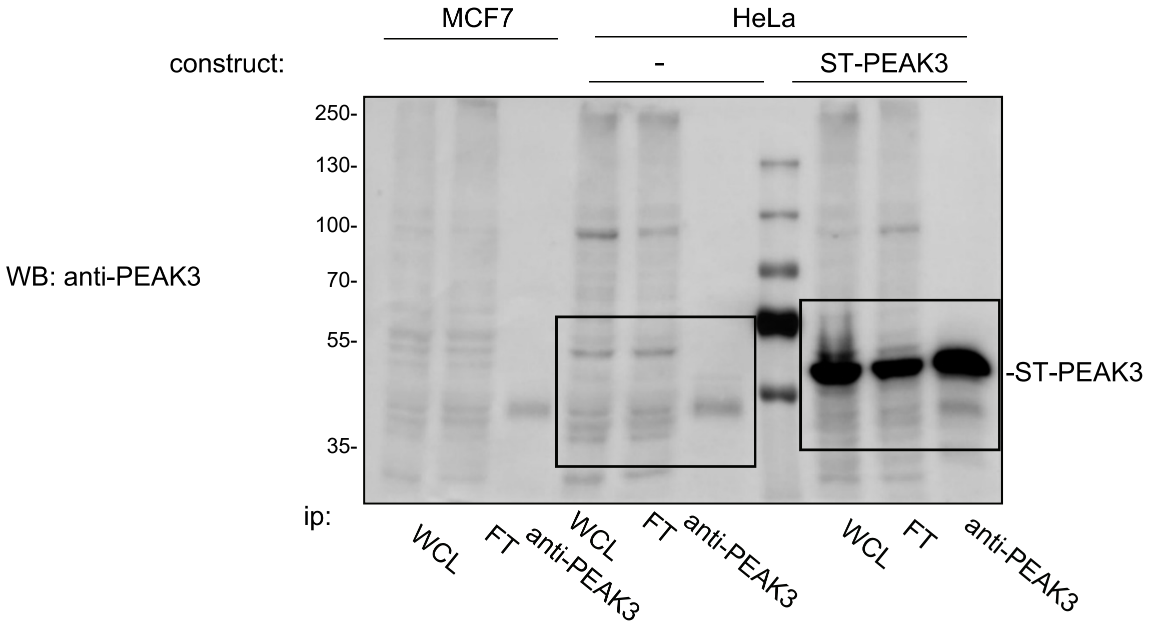

MCF7
HeLa
construct:
-
ST-PEAK3
250-
130-
100-
70-
55-
35-
WB: anti-PEAK3
-ST-PEAK3
ip:
WCL
FT
anti-PEAK3
WCL
FT
anti-PEAK3
WCL
FT
anti-PEAK3

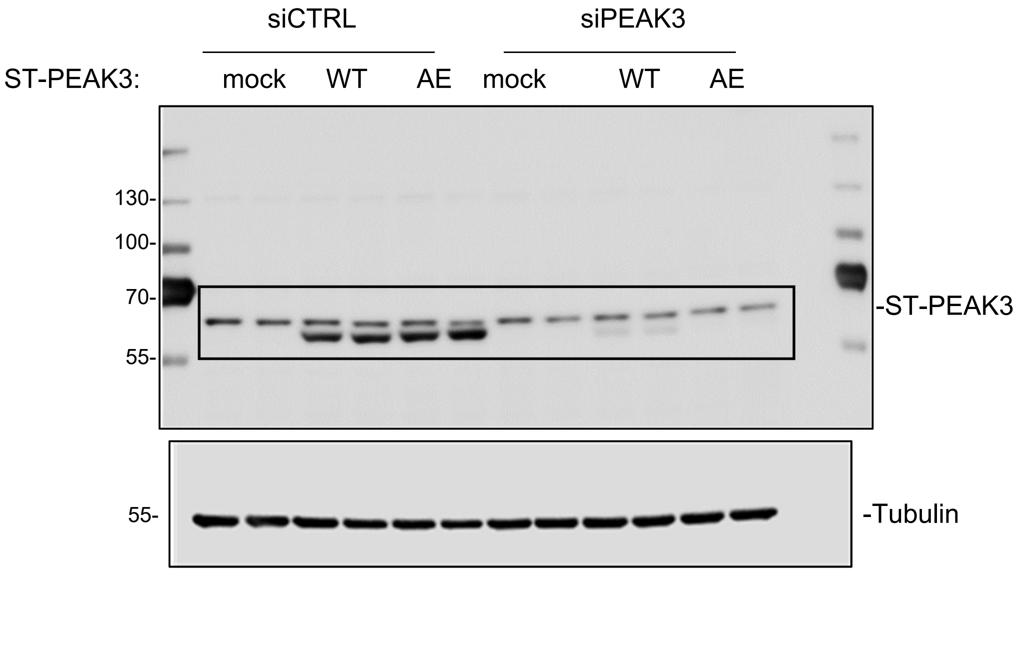

siCTRL
siPEAK3
ST-PEAK3:
mock
WT
AE
mock
WT
AE
130-
100-
70-
55-
-ST-PEAK3
55-
-Tubulin

Figure S2

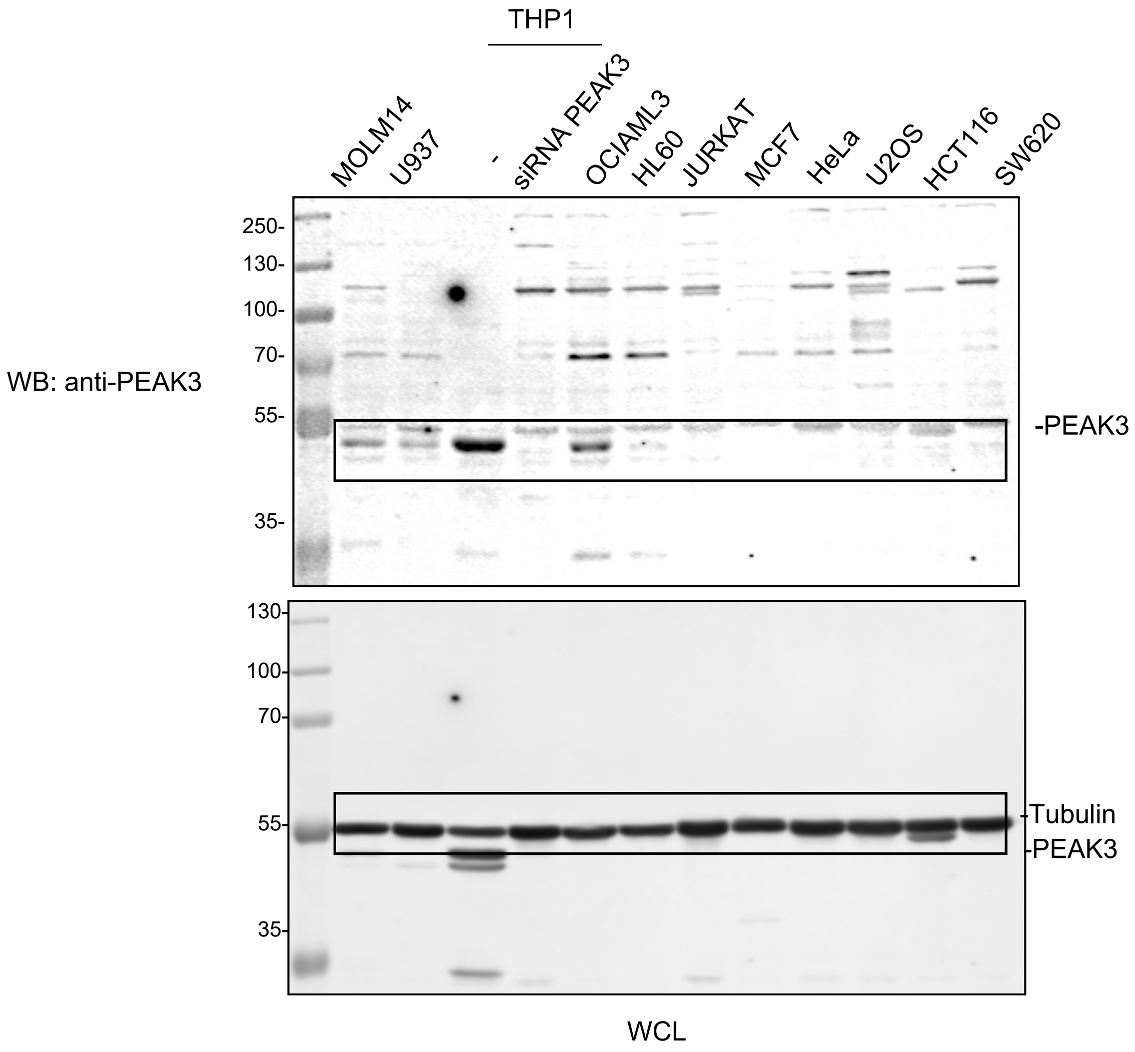
THP1
MOLM14
U937
-
siRNA PEAK3
OCIAML3
HL60
JURKAT
MCF7
HeLa
U2OS
HCT116
SW620
WB: anti-PEAK3
250-
130-
100-
70-
55-
35-
-PEAK3
130-
100-
70-
55-
35-
-Tubulin
-PEAK3
WCL

Figure S4

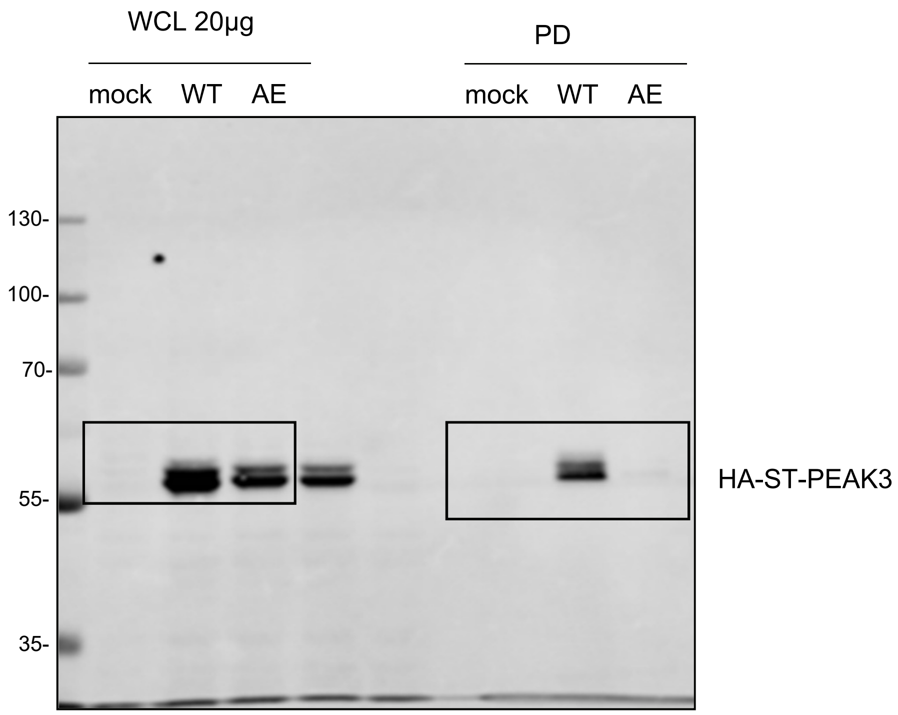
WCL 20µg
PD
mock
WT
AE
mock
WT
AE
130-
100-
70-
55-
35-
HA-ST-PEAK3

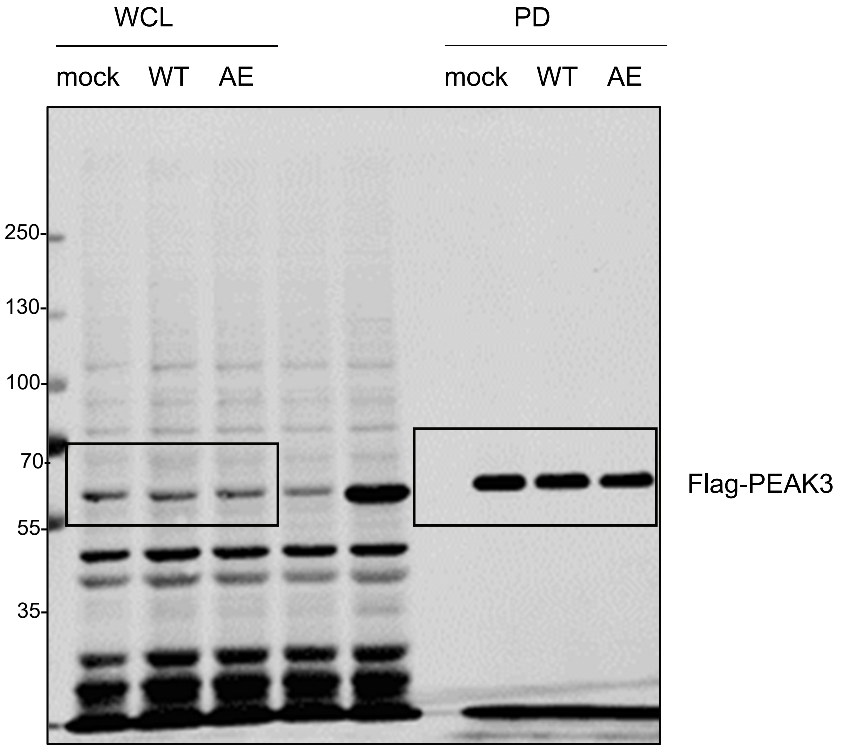
WCL
PD
mock
WT
AE
mock
WT
AE
250-
130-
100-
70-
55-
35-
Flag-PEAK3

Figure S5

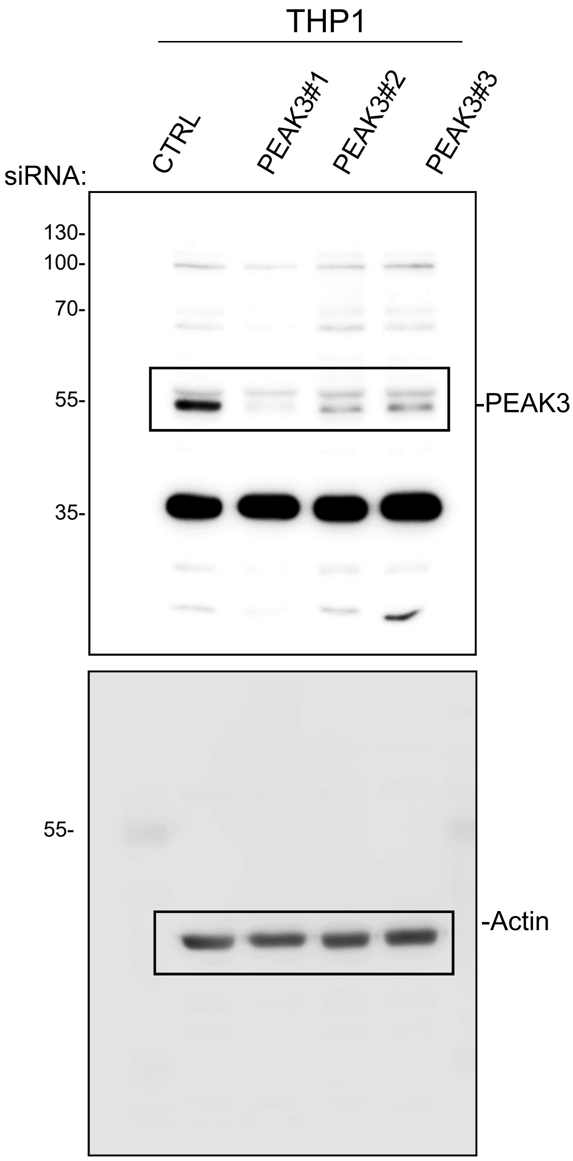
THP1
CTRL
PEAK3#1
PEAK3#2
PEAK3#3
siRNA:
130-
100-
70-
55-
35-
-PEAK3
55-
-Actin

Figure S7

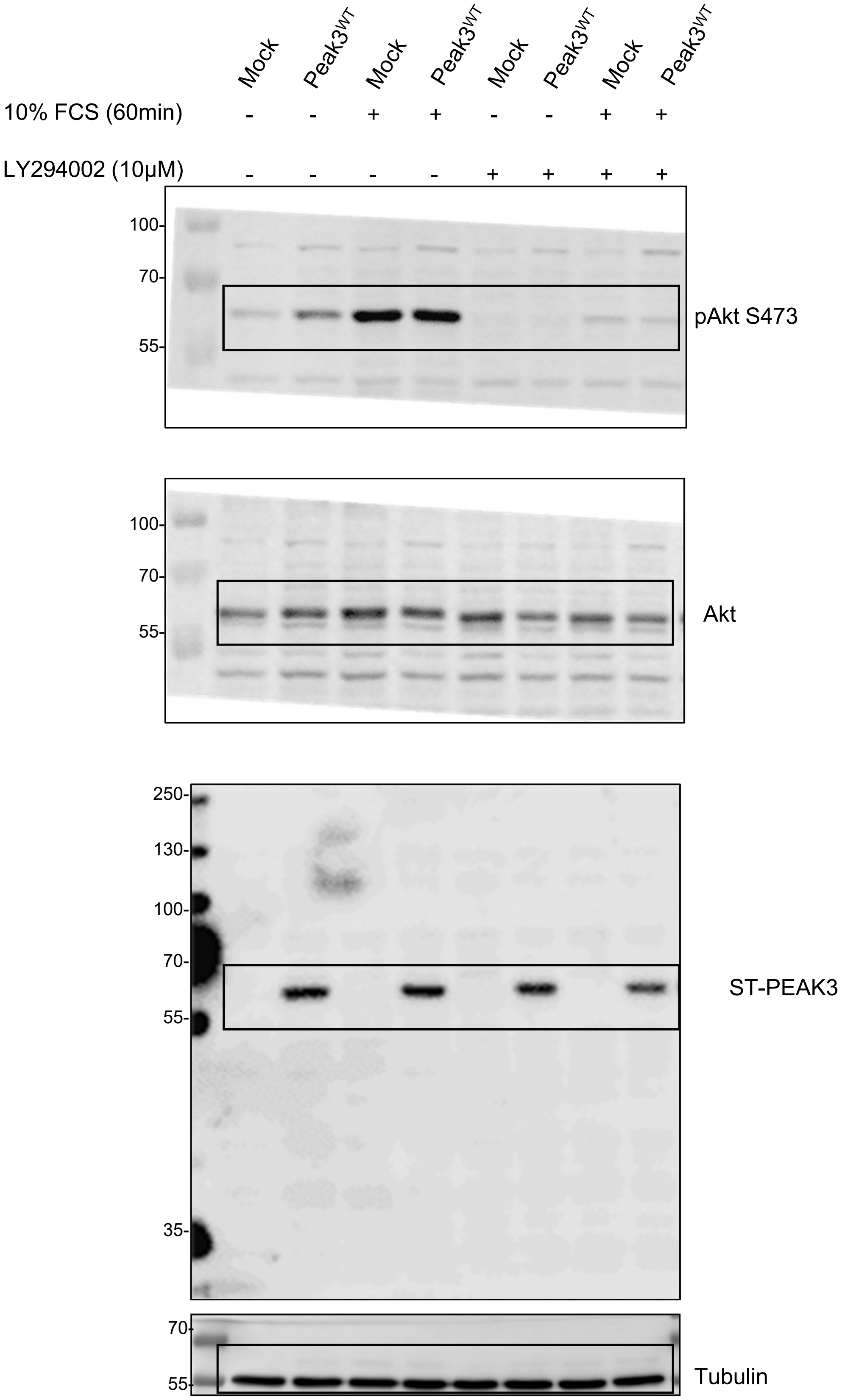

Mock
Peak3WT
Mock
Peak3WT
Mock
Peak3WT
Mock
Peak3WT
10% FCS (60min) - - + + - - + +
LY294002 (10µM) - - - - + + + +
100-
70-
55-
pAkt S473
100-
70-
55-
Akt
250-
130-
100-
70-
55-
35-
ST-PEAK3
70-
55-
Tubulin

Figure S8

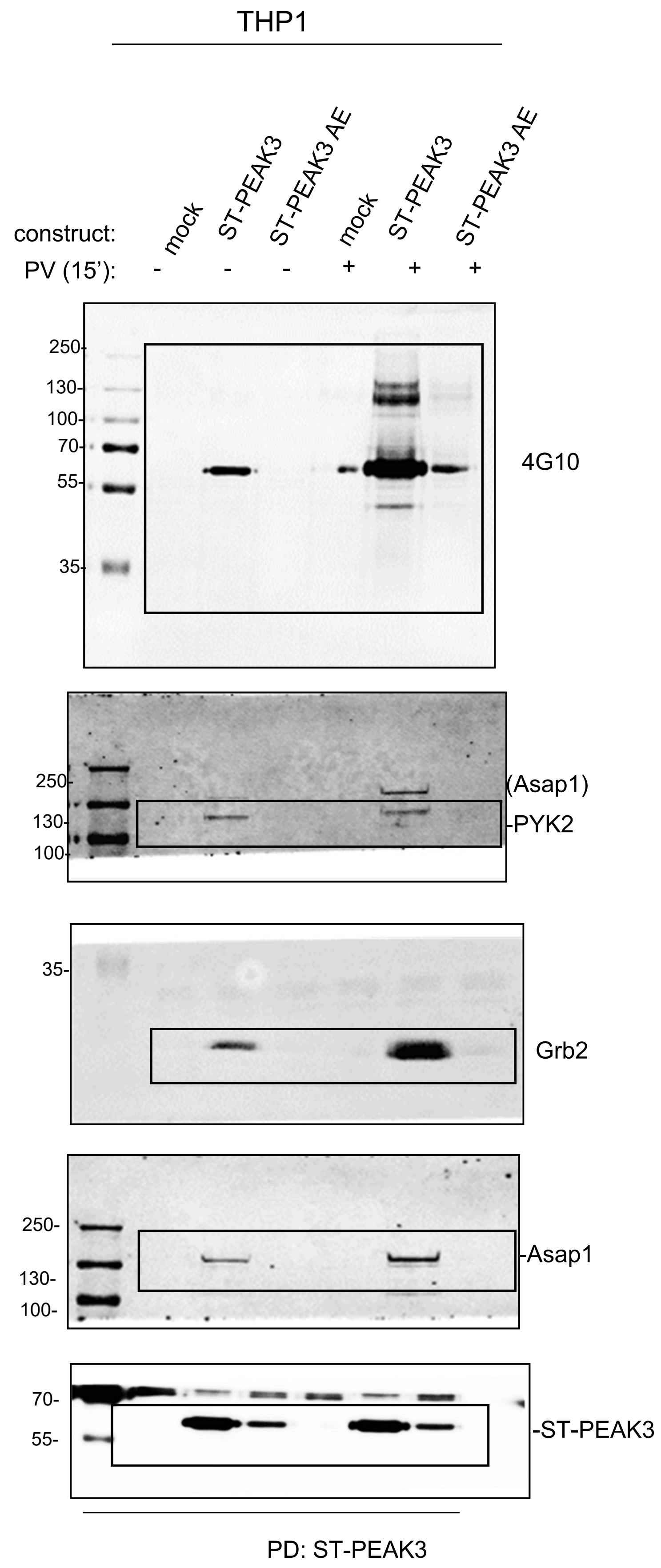
THP1
construct:
mock
ST-PEAK3
ST-PEAK3 AE
mock
ST-PEAK3
ST-PEAK3 AE
PV (15'):
-
-
-
+
+
+
250-
130-
100-
70-
55-
35-
4G10
250-
130-
100-
(Asap1)
-PYK2
35-
Grb2
250-
130-
100-
-Asap1
70-
55-
-ST-PEAK3
PD: ST-PEAK3

Figure S8

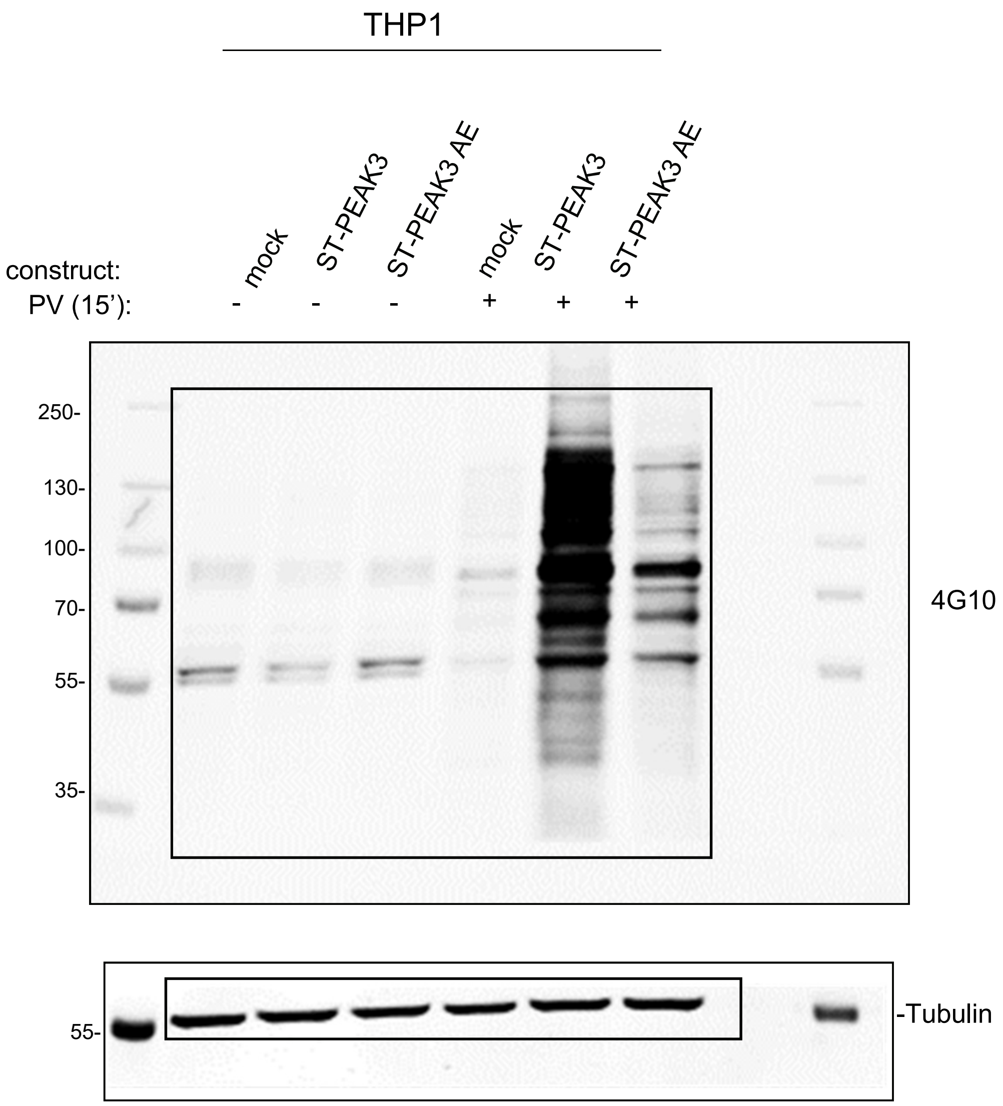
THP1
construct:
PV (15'):
mock
ST-PEAK3
ST-PEAK3 AE
mock
ST-PEAK3
ST-PEAK3 AE
-
-
-
+
+
+
250-
130-
100-
70-
55-
35-
4G10
55-
-Tubulin
