# Supplementary material for: SHED-Dependent Oncogenic Signaling of the PEAK3 Pseudo-Kinase
Source: Cancers (Basel). 2021 Dec 17;13(24):6344. doi: 10.3390/cancers13246344 (PMC8699254; doi:10.3390/cancers13246344)
Supplement: Supplementary file 1 [file cancers-13-06344-s001.zip › supplementary information/Supplementary figures.pdf]

Supplementary information

Tables S1. Accession numbers of the PEAK pseudo-kinases used in this study.

Tables S2. Characteristics of AML patient samples.

Table S3. Main hits recovered from the PEAK3 interactomic analyses in U2OS, HeLa and THP1 cells.

Figure S1. *PEAK1-3* mRNA distribution in human tissues. Normalized *PEAK1-3* expression levels from the Human Protein Atlas (24).

Figure S2. Characterization of our anti-PEAK3 antibody a. Western blot (WB) analysis with our anti-PEAK3 antibody of a total lysate of U2OS cells that overexpress indicated ST-PEAK3 construct and that was transfected with indicated siRNA. **b**. Immunoprecipitation (ip) of ST-PEAK3 overexpressed in HeLa cells with our anti-PEAK3 antibody; WCL, whole cell lysate; FT, Flow Through. **c.** Western blot analysis with our anti-PEAK3 antibody of PEAK3 expression in whole cell lysates of the indicated cell lines. The antibody specificity is shown using THP1 cells transfected with a siRNA targeting PEAK3. Both low and longer exposures are shown.

Figure S3. PEAK3 transcript level in AML patient samples. a. FAB morphology in BEAT AML. **b.** Association between PEAK3 aberrant expression and indicated specific oncogenic mutations. Is shown the mean ± SD; $^*p<0.05$; $^{**}p<0.01$ (Tukey's multiple comparisons test).

Figure S4. PEAK3 signalling in HeLa cells. a. SHED-dependent PEAK3 self-association. Co-precipitation of ST-PEAK3 (wild type, WT, and the indicated mutant) on streptavidin beads (PD, pull down) with FLAG-PEAK3 from HeLa cells that express the indicated constructs. PEAK3 expression in whole cell lysates (WCL) is also shown. Relative quantification of precipitated FLAG-PEAK3 is indicated. **b.** PEAK3 interactome in HeLa cells represented as volcano plots of the MS data.

Figure S5. siRNAs targeting PEAK3 reduce THP1 cells growth. Effect of indicated siRNAs on PEAK3 protein level and growth of THP1 cells. Is shown the mean ± SD (n=4 and 5 respectively); ***$p<0.001$ (Student's *t* test).

Figure S6. PEAK3 cellular distribution. a. Direct fluorescence of U2OS cells that express GFP-PEAK3 (WT or A436E mutant) or GFP alone. Merge: co-localization of GFP-PEAK3 with paxillin. **b**. PEAK3 localization by indirect anti-HA immunofluorescence in U2OS cells that express HA-ST-PEAK3 (wild type or the A436E mutant).

Figure S7. PEAK3-AKT signalling. a. Quantification of the selected signals from the PEAK3-dependent phospho-kinase array in THP1 cells. **b.** PEAK3 activation of AKT in U2OS cells is inhibited by incubation with the pan-PI3K inhibitor LY294002.

Figure S8. SHED-dependent PEAK3 phospho-tyrosine signalling. Pull-down of ST-PEAK3 and associated proteins from THP1 cells expressing indicated PEAK3 constructs and treated or not with pervanadate (PV) for 15min. The relative level of precipitated tyrosine phosphorylated proteins and associated PEAK3 binders is shown. The total level of protein tyrosine phosphorylation is also shown.

Figure S9. A model on PEAK3 oncogenic signalling in AML

Figure S10. Original blots used for the figures.

Figure S1

PEAK1

RNA tissue specificity: Low tissue specificity

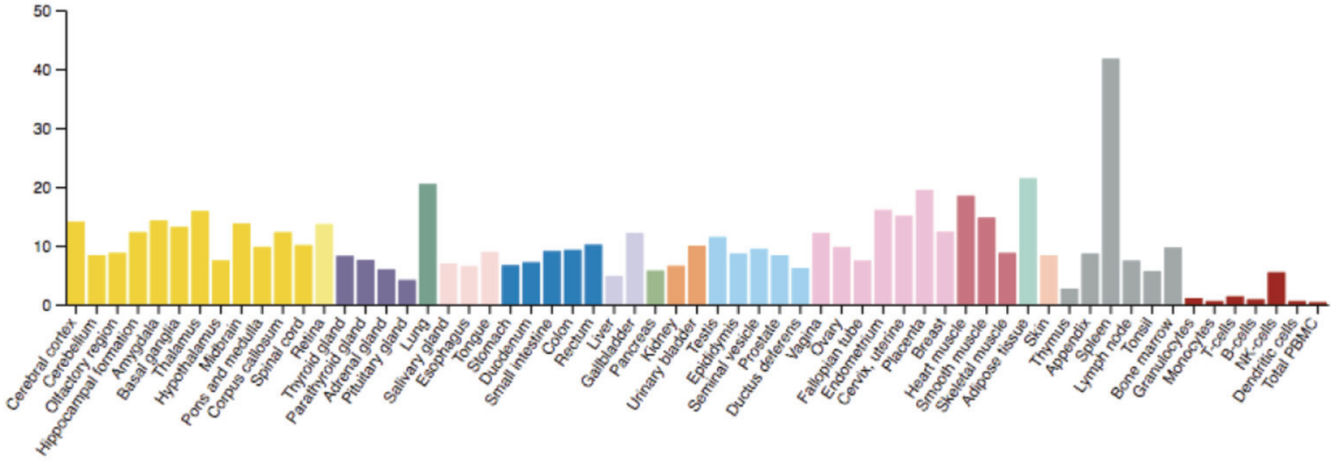

PEAK2

RNA tissue specificity: Tissue enhanced (brain)

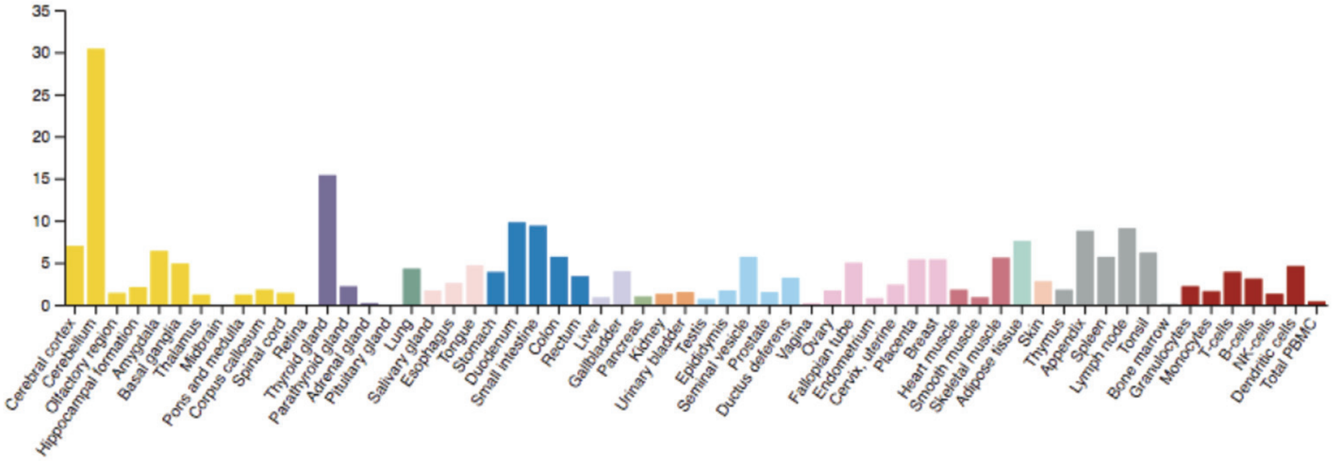

PEAK3

RNA tissue specificity: Tissue enhanced (blood, lymphoid tissue)

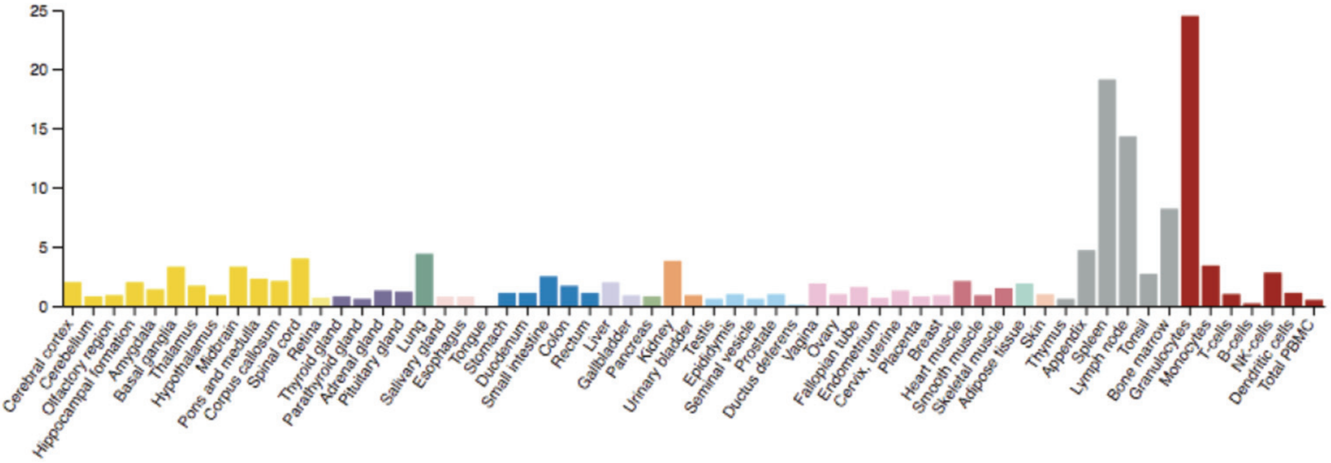

Figure S2

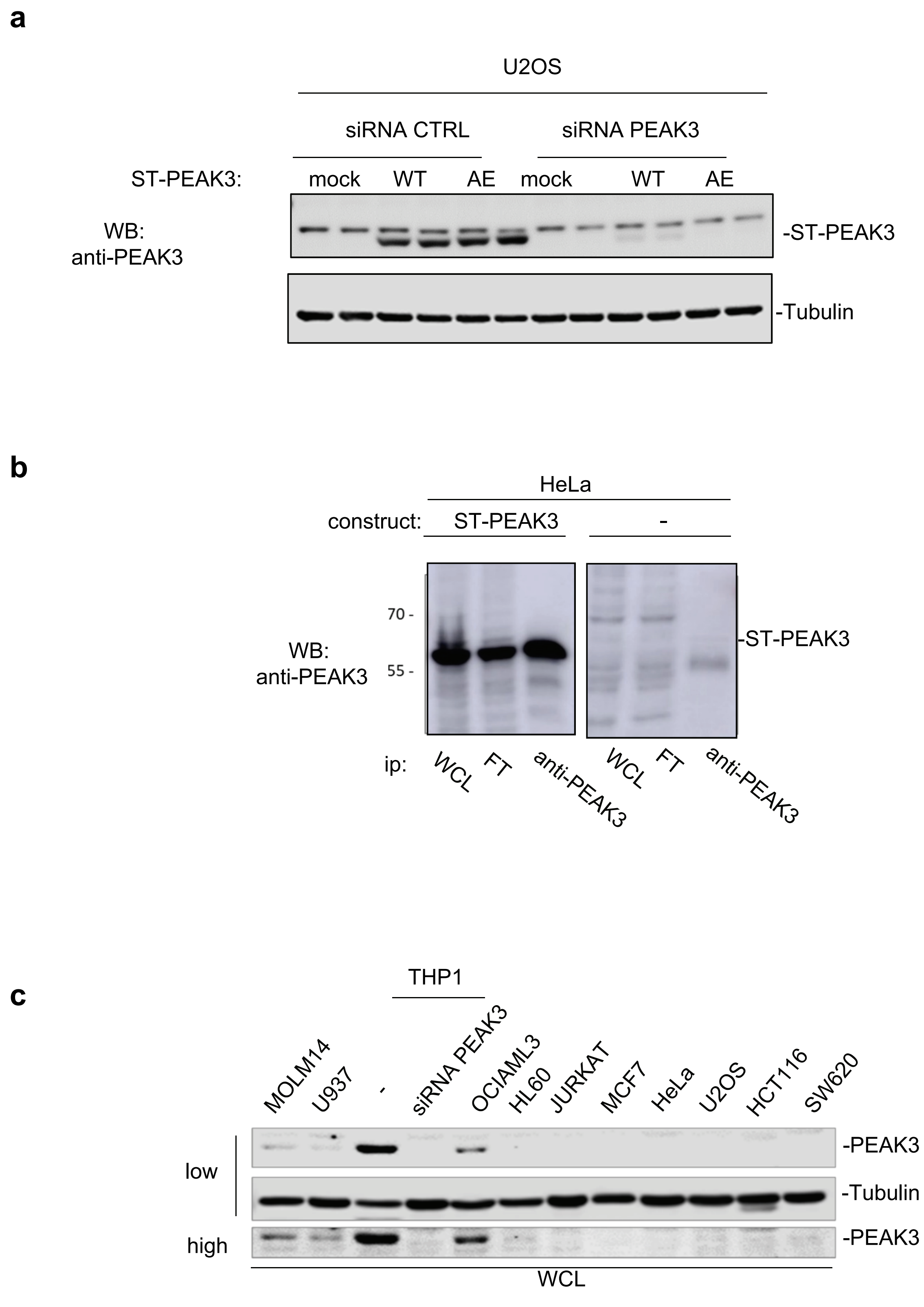
a
U2OS
siRNA CTRL
siRNA PEAK3
ST-PEAK3:
mock
WT
AE
mock
WT
AE
WB:
anti-PEAK3
-ST-PEAK3
-Tubulin
b
HeLa
construct:
ST-PEAK3
-
70 -
55 -
WB:
anti-PEAK3
-ST-PEAK3
ip:
WCL
FT
anti-PEAK3
WCL
FT
anti-PEAK3
c
THP1
MOLM14
U937
-
siRNA PEAK3
OCIAML3
HL60
JURKAT
MCF7
HeLa
U2OS
HCT116
SW620
low
high
-PEAK3
-Tubulin
-PEAK3
WCL

Figure S3

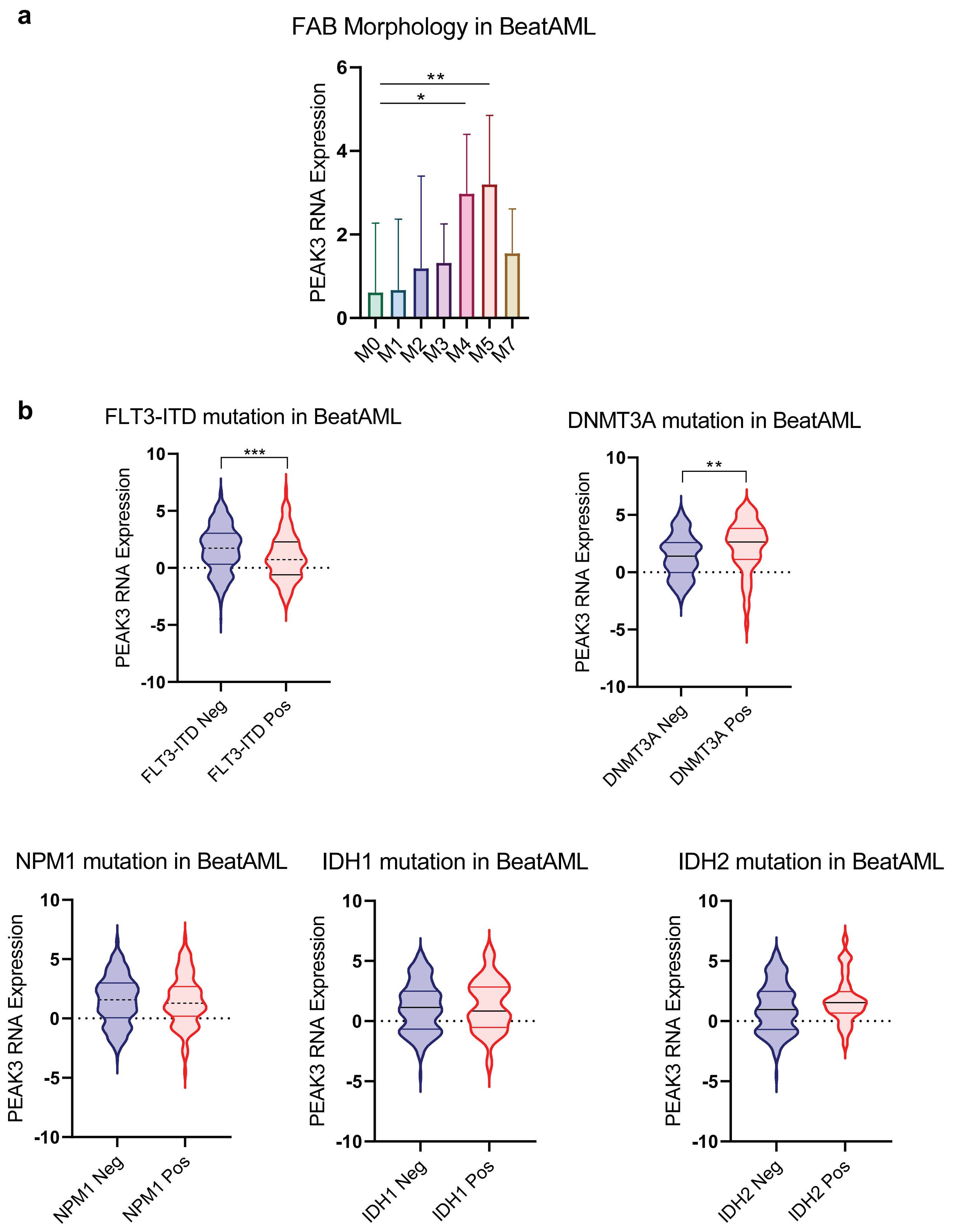
a
FAB Morphology in BeatAML
PEAK3 RNA Expression
6
4
2
0
**
*
M0
M1
M2
M3
M4
M5
M7
b
FLT3-ITD mutation in BeatAML
PEAK3 RNA Expression
10
5
0
-5
-10
***
FLT3-ITD Neg
FLT3-ITD Pos
DNMT3A mutation in BeatAML
PEAK3 RNA Expression
10
5
0
-5
-10
**
DNMT3A Neg
DNMT3A Pos
NPM1 mutation in BeatAML
PEAK3 RNA Expression
10
5
0
-5
-10
NPM1 Neg
NPM1 Pos
IDH1 mutation in BeatAML
PEAK3 RNA Expression
10
5
0
-5
-10
IDH1 Neg
IDH1 Pos
IDH2 mutation in BeatAML
PEAK3 RNA Expression
10
5
0
-5
-10
IDH2 Neg
IDH2 Pos

Figure S4

a

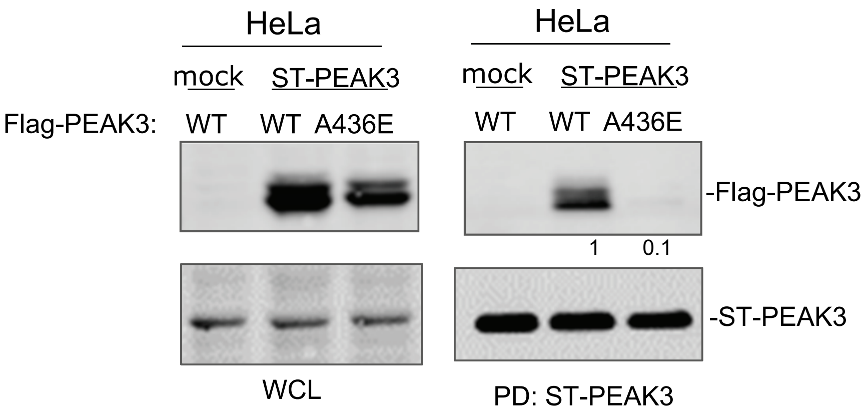

HeLa
mock
ST-PEAK3
Flag-PEAK3:
WT
WT
A436E
HeLa
mock
ST-PEAK3
WT
WT
A436E
-Flag-PEAK3
1
0.1
-ST-PEAK3
WCL
PD: ST-PEAK3

b

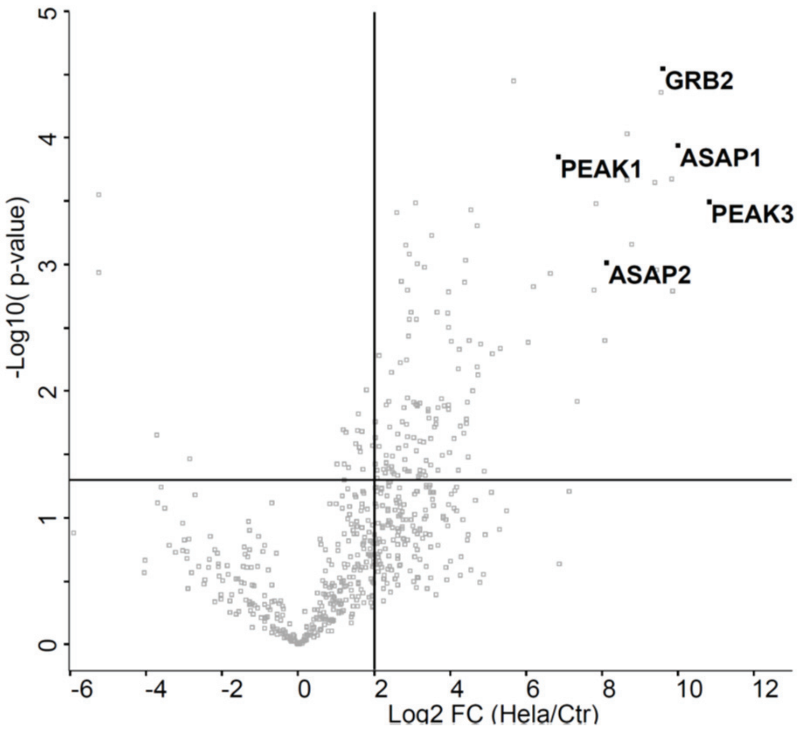

GRB2
PEAK1
ASAP1
PEAK3
ASAP2
-Log10(p-value)
Log2 FC (Hela/Ctr)

Figure S5

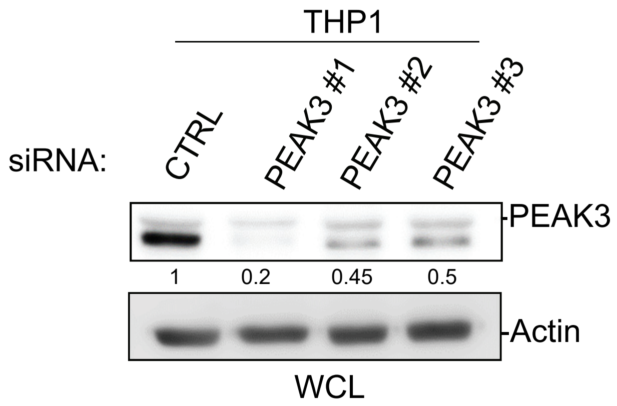
THP1
siRNA:
CTRL
PEAK3 #1
PEAK3 #2
PEAK3 #3
PEAK3
1
0.2
0.45
0.5
Actin
WCL

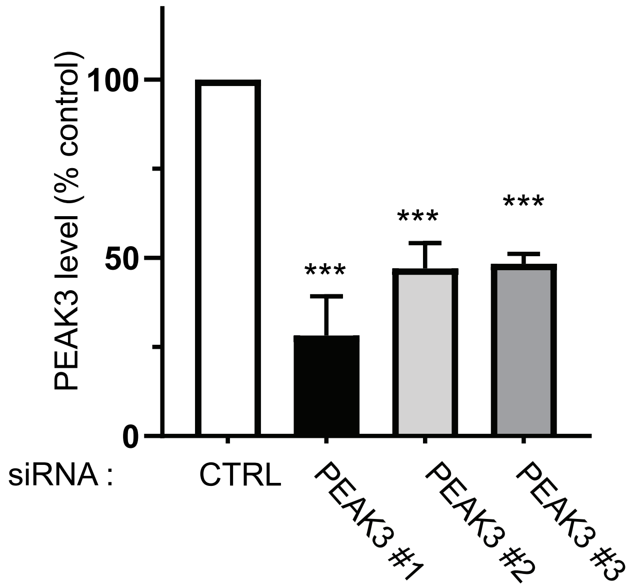
PEAK3 level (% control)
100
50
0
***
***
***
siRNA :
CTRL
PEAK3 #1
PEAK3 #2
PEAK3 #3

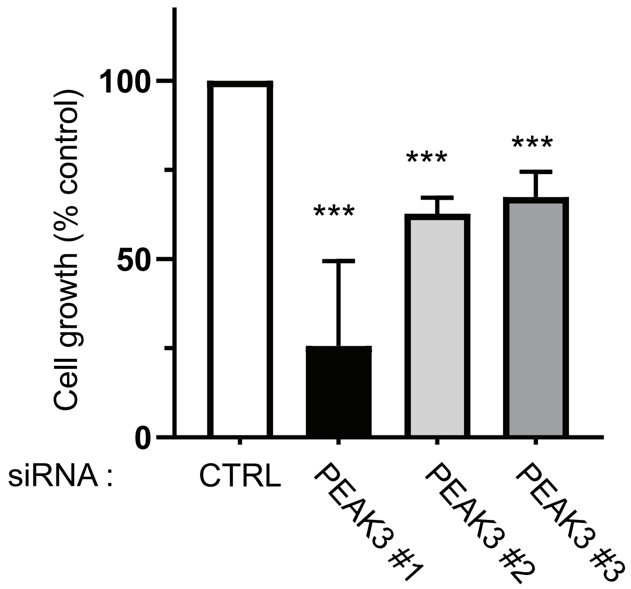
Cell growth (% control)
100
50
0
***
***
***
siRNA :
CTRL
PEAK3 #1
PEAK3 #2
PEAK3 #3

Figure S6

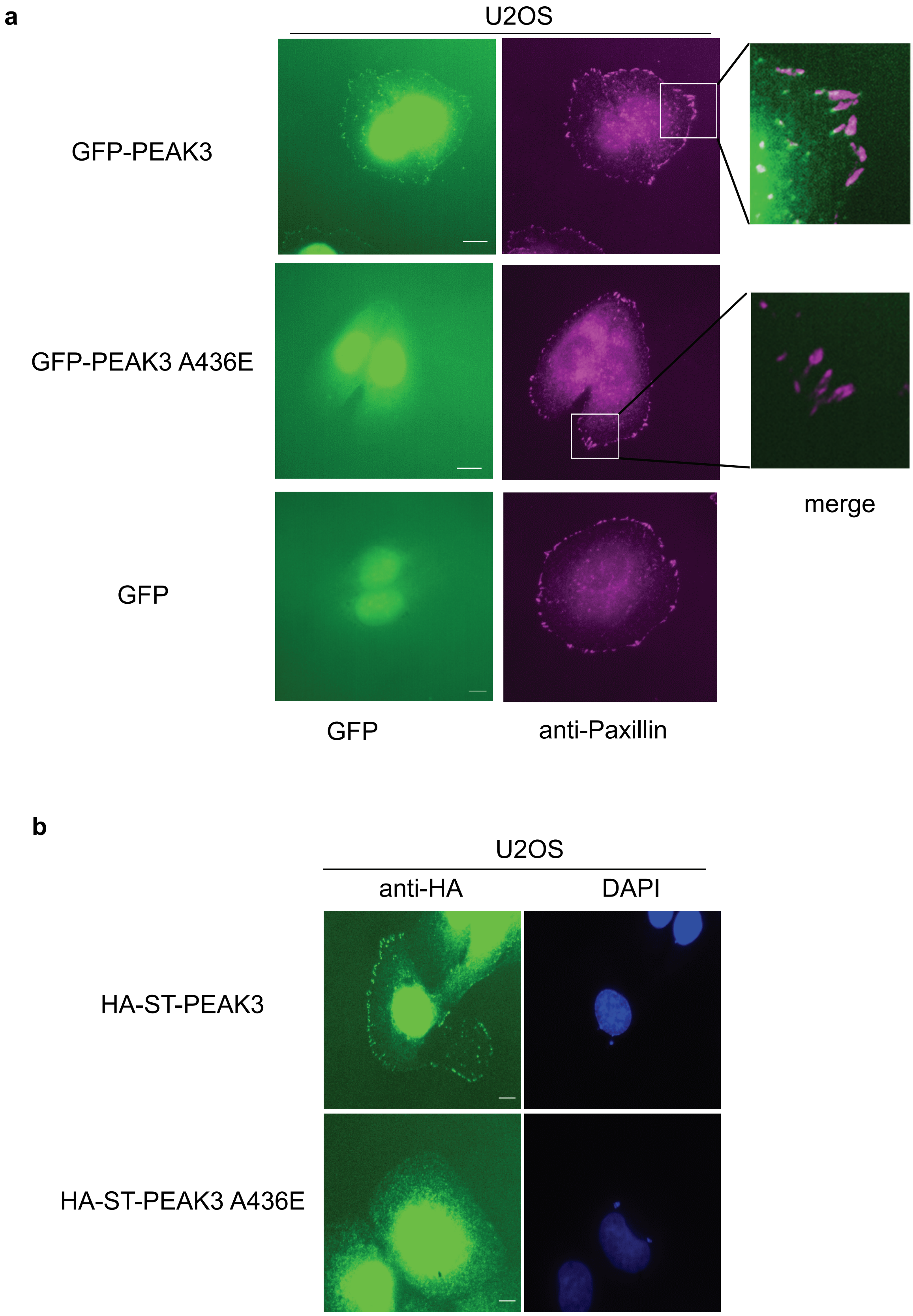
a
U2OS
GFP-PEAK3
GFP-PEAK3 A436E
GFP
merge
GFP
anti-Paxillin
b
U2OS
anti-HA
DAPI
HA-ST-PEAK3
HA-ST-PEAK3 A436E

Figure S7

a

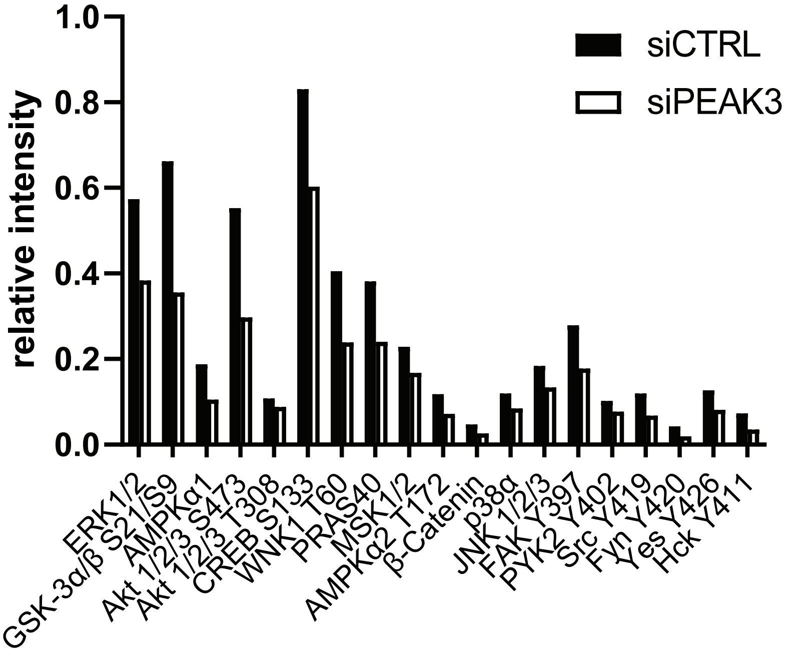

1.0
0.8
0.6
0.4
0.2
0.0
relative intensity
siCTRL
siPEAK3
ERK1/2
GSK-3α/β S21/S9
AMPKα1
Akt 1/2/3 S473
Akt 1/2/3 T308
CREB S133
WNK1 T60
PRAS40
MSK1/2
AMPKα2 T172
β-Catenin
p38α
JNK 1/2/3
FAK Y397
PYK2 Y402
Src Y419
Fyn Y420
Yes Y426
Hck Y411

b

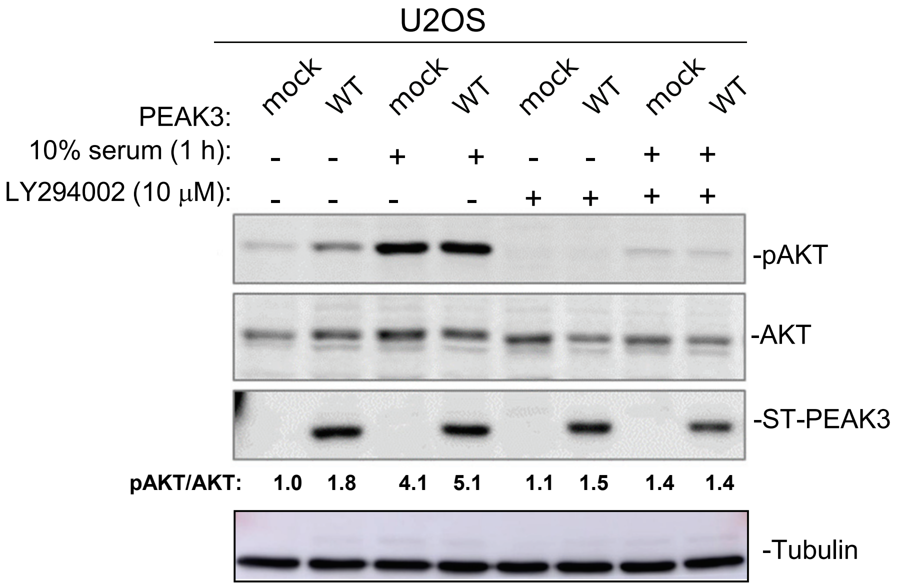

U2OS
PEAK3:
mock
WT
mock
WT
mock
WT
mock
WT
10% serum (1 h):
- - + + - - + +
LY294002 (10 μM):
- - - - + + + +
-pAKT
-AKT
-ST-PEAK3
pAKT/AKT:
1.0 1.8 4.1 5.1 1.1 1.5 1.4 1.4
-Tubulin

Figure S8

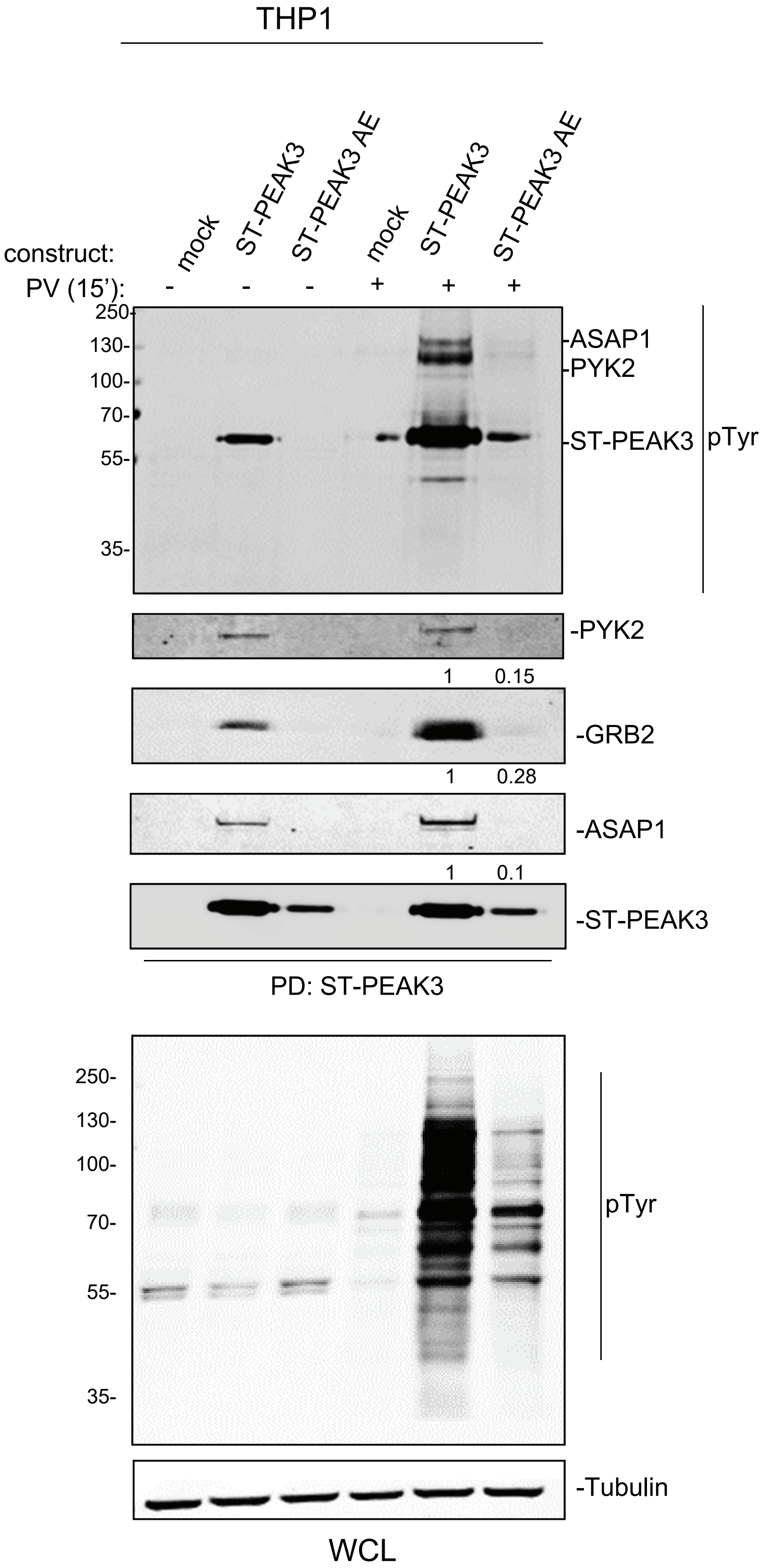
THP1
construct:
mock
ST-PEAK3
ST-PEAK3 AE
mock
ST-PEAK3
ST-PEAK3 AE
PV (15'):
-
-
-
+
+
+
250-
130-
100-
70-
55-
35-
-ASAP1
-PYK2
-ST-PEAK3
pTyr
-PYK2
1
0.15
-GRB2
1
0.28
-ASAP1
1
0.1
-ST-PEAK3
PD: ST-PEAK3
250-
130-
100-
70-
55-
35-
pTyr
-Tubulin
WCL

Figure S9

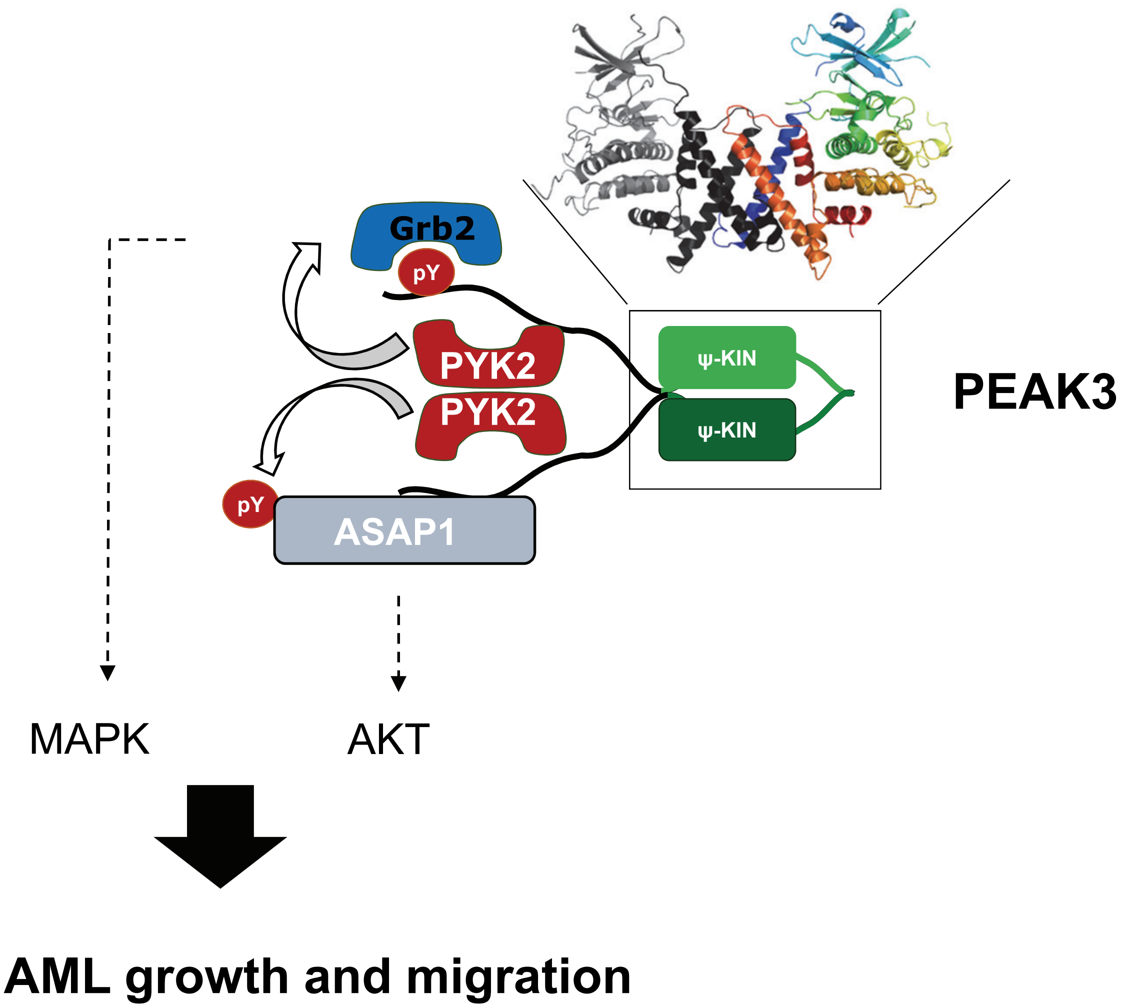
Grb2
pY
PYK2
PYK2
pY
ASAP1
ψ-KIN
ψ-KIN
PEAK3
MAPK
AKT
AML growth and migration
